# The Pro-Metastatic Roles of ROS

**DOI:** 10.3390/antiox15050529

**Published:** 2026-04-22

**Authors:** Darin E. Gilchrist, Julia A. Ju, Stuart S. Martin, Michele I. Vitolo

**Affiliations:** 1 Marlene and Stewart Greenebaum NCI Comprehensive Cancer Center, University of Maryland School of Medicine, 655 W. Baltimore St., Baltimore, MD 21201, USA; darin.glichrist@som.umaryland.edu (D.E.G.); jju@som.umaryland.edu (J.A.J.); ssmartin@som.umaryland.edu (S.S.M.); 2 Graduate Program in Molecular Medicine, University of Maryland School of Medicine, 800 W. Baltimore St., Baltimore, MD 21201, USA; 3 Medical Scientist Training Program, University of Maryland School of Medicine, 20 Penn St., RM S359, Baltimore, MD 21201, USA; 4 Department of Pharmacology and Physiology, University of Maryland School of Medicine, 655 W. Baltimore St., Baltimore, MD 21201, USA

**Keywords:** reactive oxygen species (ROS), oxidative stress, hypoxia, hydrogen peroxide, cancer, metastasis, epithelial-to-mesenchymal transition (EMT), circulating tumor cells (CTCs), anoikis, ROS detection

## Abstract

Metastasis is a complex, multistep process in which cancer spreads from its original tumor to other sites in the body. During metastasis, tumor cells move away from the primary tumor and intravasate into the lymphatics or circulation. Surviving tumor cells can then extravasate into and remain in distant tissues until they once again begin to proliferate, forming secondary tumors. An excess of reactive oxygen species (ROS) can promote metastasis, dependent on the ROS molecule, its level of excess, and the examined step within the metastatic cascade. Here, we highlight recent studies where ROS promote epithelial-to-mesenchymal transition, cell migration and invasion, circulating tumor cell survival and disseminated tumor cell dormancy. Additionally discussed are novel in vivo ROS detection methods, FDA-approved therapies and clinical trials that manipulate ROS to improve cancer patient survival. Since metastasis is the major cause of cancer-related death, a better understanding of this process and ROS as a contributing factor will help to identify novel targets for inhibition or prevention.

## 1. Introduction

Cancer is broadly defined as a group of diseases characterized by the uncontrolled growth and division of abnormal cells with the capacity to invade surrounding tissues and metastasize to distant parts of the body. Before a more advanced understanding of cancer, early successes in cancer therapy involved agents that reliably inhibited or killed actively dividing cells with the major goal of shrinking the cancerous tumor. While cancer detection methods and therapies are consistently improving, the focus of cancer treatment continues to be inhibiting proliferation and killing tumor cells. Cell proliferation is more straightforward and better understood, easier to drug, and produces rapid, measurable effects, whereas metastasis is still a major challenge due to its complex biology, long timeframe and incomplete mechanistic knowledge. However, with over 80–90% of cancer deaths being due to metastatic disease rather than from complications caused directly by the primary tumor [1,2], elucidating the multifaceted, dynamic mechanisms underlying metastasis is a critical need for the development of novel anti-metastatic strategies.

## 2. Oxidation–Reduction Regulation

Cellular oxidation–reduction (redox) reactions occur in tandem, in which electrons are transferred between chemical species. There are many different types of redox reactions involving different reactive species, each with unique chemical characteristics and signaling roles and all fundamental to life processes. The type of reactive species depends upon its components and molecular characteristics. There are reactive oxygen (ROS), nitrogen (RNS), sulfur (RSS), and electrophile (RES) species, all of which are oxidants, or chemical species which can accept electrons. Cells in “redox homeostasis” operate within a flexible homeodynamic space, a bounded but adjustable range of redox potentials that shifts with differentiation, stress, and disease yet is actively kept within viability-supporting limits by antioxidant systems and feedback controls [3,4,5,6]. Within this homeodynamic space, transient, localized oxidizing shifts encode information via reversible cysteine modifications on proteins [6]. In addition to metabolic regulation, other major cellular processes, including epigenetics, transcription, protein homeostasis, proliferation and differentiation, and cell death, are all under redox regulation. In biology, “oxidative stress” typically refers to an excess of oxidants over antioxidant capacity. Excessive or prolonged oxidation pushes the system outside of its bounded range, leading to oxidative stress, which can damage macromolecules and alter cell signaling, disease progression or cell death [7].

### 2.1. Intracellular ROS Production

ROS comprise a range of molecules with oxidizing properties that are formed primarily through successive one-electron reduction steps of molecular oxygen during normal physiological and pathological processes [8]. Thus, ROS participate in important cellular signaling and homeostatic functions but also cause oxidative damage if uncontrolled. Free-radical ROS include superoxide anions (O_2_*^-^), hydroxyl radicals (OH*), nitric oxide (*NO), and lipid radicals. Other ROS molecules with oxidizing properties that are not free radicals include hydrogen peroxide (H_2_O_2_), singlet oxygen (^1^O_2_), peroxynitrite (ONOO^-^), and hypochlorous acid (HOCl).

The three primary ROS species are O_2_*^-^, H_2_O_2_, and OH*, all produced from stepwise reduction of molecular O_2_ (Reaction (1)) [8]. Mitochondria are the principal organelles that generate intracellular ROS. During oxidative phosphorylation and ATP production, molecular oxygen (O_2_) is reduced to water by the electron transport chain. Complexes I and III within the electron transport chain form and release superoxide anions into the mitochondrial matrix and inner membrane as well as the cytosol [9]. Complex II can also directly or indirectly be a significant source of ROS. Superoxide anions and hydrogen peroxide are produced during the forward reaction, in which succinate is oxidized to fumarate, but superoxide anions can also be generated indirectly by reverse electron transfer (RET) from Complex II to I [10]. While not a direct component of the electron transport chain but located in the mitochondrial matrix, the multisubunit enzyme, α-ketoglutarate dehydrogenase complex (KGDHC), predominately produces superoxide, which is then converted to hydrogen peroxide dependent on the redox state and chemistry of FAD or NADH and O_2_ accessibility [11]. Other organelles, including the peroxisomes and the endoplasmic reticulum (ER), also minorly contribute to superoxide generation via the P450 system. In addition to organelles, the family of transmembrane NADPH oxidases (NOXs) transfer electrons through biological membranes, resulting in oxygen reduction and generating superoxide (NOX1-3 and NOX5) [12].

(1) $$O_{2}\overset{{+e}^{-}}{\to }O_{2}^{*-}\overset{{+e}^{-}({+2H}^{+})}{\to }H_{2}O_{2}\overset{{+e}^{-}}{\to }{\mathrm{HO}}^{*}{+\mathrm{HO}}^{-}\overset{{+e}^{-}({+2H}^{+})}{\to }{2H}_{2}O$$

Small amounts of hydrogen peroxide can act as a second messenger, regulating redox signaling to maintain homeostatic levels. Unlike superoxide, hydrogen peroxide can passively diffuse through the plasma membrane or make its way into the cell via channel proteins called aquaporins. Hydrogen peroxide can also be generated intracellularly by the dismutation of superoxide, which may occur spontaneously or via superoxide dismutase (SOD) (Reaction (2)) [13]. SODs (SOD1-3) are enzymatic antioxidants that can diffuse between cellular compartments and appear in the cytosol, mitochondria, nucleus or extracellular space to catalyze superoxide radicals into hydrogen peroxide and molecular oxygen. Several other oxidases, including peroxisomal oxidases and monoamine oxidases on the outer mitochondrial membrane, can also convert superoxide into hydrogen peroxide. The organelles responsible for hydrogen peroxide generation are peroxisomes and ER. Peroxisomes generate large amounts of hydrogen peroxide via oxidases involved in fatty-acid β-oxidation and other catabolic pathways, while the ER produces hydrogen peroxide during oxidative protein folding and through the ER-localized NOX4 isoform. Although hydrogen peroxide is comparatively less reactive than superoxide, its accumulation is damaging. Enzymes such as catalases (CAT) and glutathione peroxidases (GPx) can break down excess hydrogen peroxide to water and oxygen [14].

(2) $${2O}_{2}^{*-}{+2H}^{+}\overset{\mathrm{SOD}}{\to }H_{2}O_{2}{+O}_{2}$$

The hydroxyl radical is by far the most reactive of the three primary ROS species. It can react indiscriminately with nearly any nearby molecule and has a strong affinity for aromatic or sulfur-containing molecules rich in electrons such as proteins and DNA [15]. The hydroxyl radical is formed within the cell as an unintended side reaction while trying to eliminate superoxide and hydrogen peroxide following Fenton/Haber–Weiss chemistry when in the presence of redox-active metals, particularly iron. This occurs following two steps, where the Haber–Weiss reaction (Reaction (3)) is followed by a second step, termed the Fenton reaction (Reaction (4)), yielding a net reaction (Reaction (5)).

(3) $${\mathrm{Fe}}^{3+}+O_{2}^{*-}\to {\mathrm{Fe}}^{2+}+O_{2}$$

(4) $${\mathrm{Fe}}^{2+}+H_{2}O_{2}\to {\mathrm{Fe}}^{3+}+{\mathrm{HO}}^{*}+{\mathrm{HO}}^{-}$$

Net reaction:

(5) $$O_{2}^{*-}+H_{2}O_{2}\to {\mathrm{HO}}^{*}+{\mathrm{HO}}^{-}+O_{2}$$

### 2.2. Pathological ROS Levels/Activity

Balanced redox reactions and controlled ROS production are central to energy generation and metabolism, biosynthesis and anabolic pathways, antioxidant defense and detoxification, redox signaling and regulation, and immunity [8,16,17,18,19,20,21,22,23]. Moderate levels of ROS within a homeodynamic range act as important signals for cell growth and differentiation; however, deviations outside the range are detrimental to the cell. Since the direction of redox reactions in aerobic cell metabolism is towards oxidation, high levels of reductants or antioxidants (reductive stress) are considered subphysiological, and high levels of oxidants or low antioxidant levels (oxidative stress) are considered supraphysiological [4]. Both reductive and oxidative stress are forms of redox stress, and, although via differing mechanisms, they can damage all major biomolecules, trigger multiple forms of cell death, and contribute to many chronic diseases. Table 1 lists the main enzymatic sources, steady-state levels, approximate lifetimes, and dominant signaling roles of ROS and damaging effects from oxidative stress on different biomolecules from the three primary ROS species [8,24,25,26]. Characteristics for the remaining five ROS species can be found in the Supplementary Table S1 [8,26,27,28,29,30].

**Figure 1:**
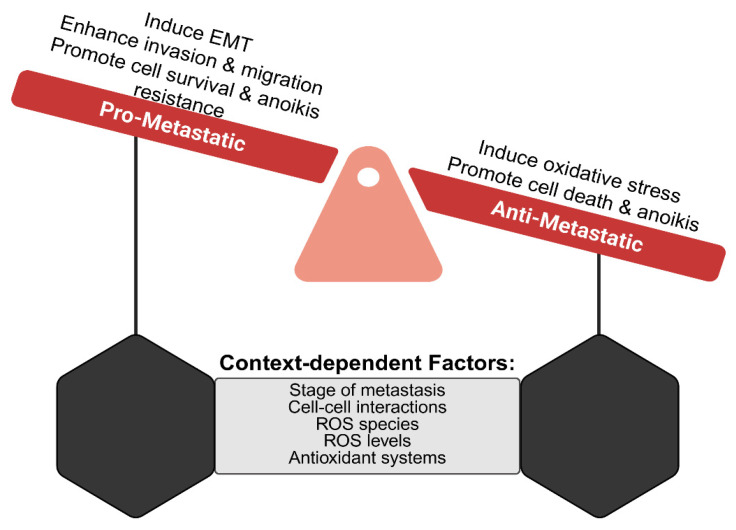
A shift in the balance between antioxidant or prooxidant response has the potential to promote or inhibit metastasis. ROS production and oxidative stress can promote or inhibit metastasis. The effect of ROS is dependent on multiple factors such as stage of metastasis (i.e., cell migration, CTC survival, DTC dormancy, etc.), cell–cell interaction (homotypic clustering, cell association with immune cells, etc.), the ROS species, ROS levels and the antioxidant system. Isolating the specific effects of different species of ROS during metastasis is very difficult due to multiple contributing factors. Created in BioRender. Gilchrist, D.E. (2026) https://BioRender.com/c2448457 (accessed on 25 March 2026).

ROS act as a central hub for cell signaling to multiple cell death programs, depending on the context, intensity, and subcellular site of ROS generation. Cells unable to counter excessive ROS to rebalance their redox reactions may trigger any one of the following modes of program cell death, collectively known as oxidative cell death: apoptosis, ferroptosis, pyroptosis, paraptosis, parthanatos, oxeiptosis, necroptosis, or autophagy-dependent cell death [31,32]. Survival and/or continued proliferation of ROS-damaged cells are associated with inflammatory and age-related conditions, cardiovascular, metabolic, neurodegenerative diseases and cancer [32,33,34,35,36,37,38,39]. Focusing on cancer, ROS are involved in all stages of cancer progression; however, their role and impact on metastasis depends on tumor type, ROS species, site of generation and levels, and counteracting antioxidant or prooxidant response (Figure 1).

## 3. The Metastatic Cascade

**Figure 2:**
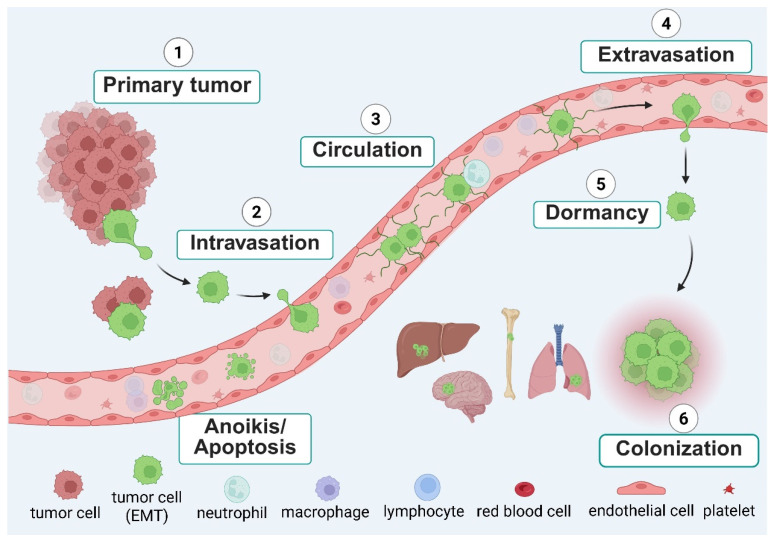
The metastatic cascade. Primary tumor cells that acquire more migratory and invasive characteristics via EMT can move away from neighboring cells and intravasate into the bloodstream. Most tumor cells that enter the bloodstream die due to detachment-induced death (anoikis) or fragmentation from external forces. Surviving circulating tumor cells (CTCs) may extravasate through the endothelial cell wall of blood vessels and remain dormant in distant tissues until they proliferate to form overt secondary tumors. CTC clusters made up of 2 or more tumor cells (green) are predicted to have initially moved away from the primary tumor together rather than associating in the bloodstream. Heterotypic clusters (green and pink) made up of tumor and immune cells may either migrate together from the initial tumor or associate with each other while in the bloodstream depending on the associating immune cell(s). Created in BioRender. Ju, J.A. (2026) https://BioRender.com/c2448457 (accessed on 25 March 2026).

Cancer metastasis is a complex, multistep process in which cells disseminate from the initial tumor and relocate to other tissues within the body. Disseminating tumor cells (DTCs) invade the neighboring tissue, intravasate into the bloodstream and/or lymphatics, reattach to vessel walls and then extravasate into distant tissues. After extravasation, the DTCs may either remain dormant or continue to proliferate into secondary tumors (Figure 2). This metastatic process is mediated by cancer cell intrinsic properties, such as genetic mutations, epigenetic modifications, altered signaling pathways, metabolic reprograming, and/or mechanisms for immune evasion fundamentally driving tumor cell behavior [40,41]. In this section, we will briefly discuss the process of epithelial-to-mesenchymal transition (EMT), which promotes tumor cell dissemination and disseminating versus circulating tumor cells.

### 3.1. Epithelial-to-Mesenchymal Transition

Approximately 85% of all malignant tumors in adults are carcinomas, arising from epithelial cells which have lost their specialized tissue barrier function and instead regain cellular plasticity by undergoing an epithelial-to-mesenchymal transition (EMT) [42]. EMT is an essential process in embryonic development and tissue repair, but the process is hijacked by cancer cells during tumor progression and metastasis [43,44]. While an EMT may not be essential for every epithelial-derived cancer to metastasize, those undergoing EMT gradually lose their epithelial characteristics such as polarity and adhesion and acquire more migratory and invasive capacity similar to that of mesenchymal cells [45]. Some canonical markers for EMT changes are the loss of E-cadherin and/or the gain of vimentin, fibronectin, and N-cadherin [46]. However, simply the absence of epithelial or presence of mesenchymal markers does not represent the spectrum of EMT-associated cell behaviors. Cells with a mixture of epithelial and mesenchymal markers (partial EMT) or those with reduced epithelial and gains in mesenchymal markers (intermediate EMT) continue to maintain plasticity and are able to switch between both cell states, fueling metastasis [47,48,49,50]. However, cells which have completely shifted to a mesenchymal phenotype (extreme EMT) with only mesenchymal markers maintain their highly invasive phenotypes but are less metastatic due to their lack of plasticity and inability to reactivate proliferation after reaching distant organs [47].

### 3.2. Disseminating Tumor Cells

In addition to acquiring migratory and invasive characteristics, DTCs also need to navigate inhospitable, changing microenvironments and host factors, and thus, most do not survive [51,52]. While metastatic efficiency can vary by cancer type, organ, and additional factors, estimates from the experimental and clinical literature suggest that less than 0.01–0.02% of all DTCs eventually outgrow into overt metastases [53]. Most either die, remain dormant, or are eliminated by host defenses, meaning that the vast majority of DTCs never successfully colonize distant organs [54,55,56,57].

For tumors to grow past 1–2 mm^3^, the threshold of nutrient and oxygen diffusion, they need vascular support [58,59]. Tumor cells produce stimulating factors to hijack the normal biological process of angiogenesis, recruiting endothelial cells from existing blood vessels to form new vessels into the tumor, allowing for sustained oxygen and nutrient delivery. However, these new vessels are leaky, primarily due to abnormal and incomplete maturation, allowing for actively invading tumor cells to more easily make their way into the bloodstream [60,61]. In some aggressive cancers, the tumor cells themselves acquire the ability to form channel-like structures that conduct blood or other fluids. This phenomenon is called vascular mimicry and occurs independently of endothelial cell recruitment from pre-existing blood vessels [62]. Since these mimicked channels may either transport blood or directly connect with endothelial-lined vessels, tumor cells lining these structures are highly exposed to circulating blood flow. As a result, they are more likely to detach and enter the circulation [62,63,64]. Thus, mosaic vessels, where both tumor cells and endothelial cells form parts of the blood vessel wall, can act as direct bridges, allowing tumor cells to transition from tumor tissue into circulation, effectively enhancing tumor cell shedding into the bloodstream.

Even small tumors can shed millions of cells into the bloodstream daily. Based on experimental measurements across different tumor models, a general estimate of the number of cells able to enter the bloodstream from a 1 cm^3^ (~1 g) small, vascularized solid tumor is approximately 1 million cells per day, although shedding rates can vary by tumor type, location, vascularity, and microenvironment [65,66]. Thus, metastatic spread may occur simultaneously and in parallel to primary tumor growth, not as a later, progressive event [67]. Strong evidence from clinical, genomic, and mathematical modeling studies all support the fact that metastasis can, and typically does, occur before a primary tumor is detectable by conventional imaging techniques [68,69].

### 3.3. Circulating Tumor Cells

Circulating tumor cells (CTCs) are DTCs that have entered the bloodstream or lymphatics. From experimental modeling, mouse and human data, the lifetime of a CTC in the bloodstream is limited to only minutes to a few hours [70]. Besides CTCs surviving anoikis, a specific type of apoptosis triggered by extracellular matrix detachment, CTCs need to survive immune cell clearance, blood sheer stress, and fragmentation due to size constraints from smaller capillary vessels [71]. Approximately only 0.01–0.1% of CTCs survive this hostile microenvironment to form tumors at a secondary site, and this is thus considered the rate-limiting step of metastasis [72,73].

**Figure 3:**
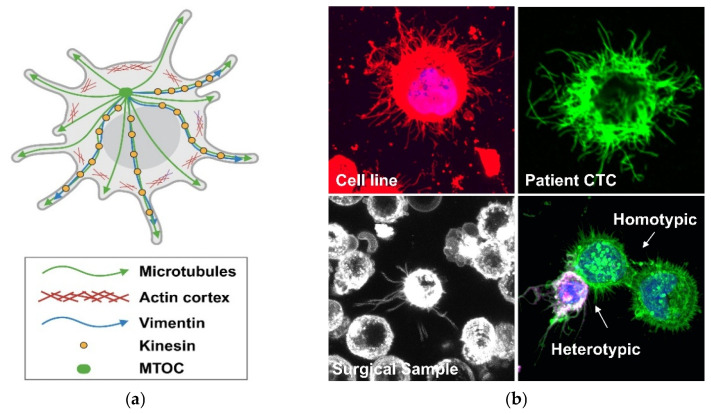
Microtentacles (McTNs) are tubulin-driven protrusions of the membrane on detached cells which aid in cell–cell association. (**a**) Diagram of a detached cell producing McTNs. McTNs are promoted by tubulin posttranslational modification indicative of microtubule stability, weakening of the actin cortex, and association of vimentin, tau and/or kinesins. Created in BioRender. Ju, J.A. (2026) https://BioRender.com/c2448457 (accessed on 25 March 2026). (**b**) I. McTNs produced by the BT549 breast cancer cell line. Suspended cells were immobilized on the TetherChip, fixed and stained with WGA-594 (red, cell membrane) and DAPI (blue, nucleus) The cell body is overexposed to better visualize the McTNs. II. A CTC producing McTNs isolated from the blood of a breast cancer patient immobilized on the TetherChip, fixed and stained with α-tubulin and alexa-488 (green) antibodies. III. Dissociated tumor cells from a patient’s biopsy producing McTNs. This image is a representative frame from a 5 min movie of live cells (binary image in black and white). McTNs are dynamic cell protrusions and were moving in and out of the z-stack range. IV. McTNs during homotypic clustering of MDA-MB-231 breast cancer cells expressing GFP-membrane (green) and heterotypic cell clustering between an MDA-MB-231 breast cancer cell and a differentiated neutrophil (white). Clustered cells are immobilized on the TetherChip, fixed and stained. Neutrophil (white) identified with an anti-CD11b antibody (magenta) and WGA-488 (green) to stain the cell membrane. The overlay of the magenta and green channels creates the white cast. DAPI was used to visualize nuclei (blue). Images were taken with a 60× objective on either an Olympus IX81 microscope with a Fluoview FV1000 confocal laser scanning system (Olympus Corporation, Center Valley, PA, USA) or a Nikon Ti2-E inverted microscope with a Nikon AX-R confocal system (Nikon Instruments Inc., Tokyo, Japan).

Most CTCs found in patient or mouse models are found as single cells, but CTC clusters have been detected. CTC clusters, sometimes referred to as circulating tumor microemboli, circulating micrometastases or circulating tumor cell aggregates, are defined by having two or more cells grouped together in a “cluster” [74,75,76]. Clusters are of clinical importance since CTC clusters are 50–100 times more likely than individual cells to survive dissemination and cause metastatic outgrowth [75,77,78]. CTC clusters in the bloodstream may be either homotypic or heterotypic clusters. Homotypic clusters are composed of only tumor cells, while heterotypic clusters can include tumor cell interactions with surrounding immune or stromal cells in the blood vasculature. Our group has identified microtentacles (McTNs), tubulin-driven membrane protrusions produced from detached cancer cell lines, dissociated tumor cells from surgical samples, and CTCs isolated from patients’ blood [79,80] (Figure 3). McTNs promote cell clustering, endothelial reattachment and CTC retention in distant tissues during metastasis [81,82,83,84,85,86,87,88,89,90] and may be a mechanism for initial heterotypic cell clustering and/or reattachment to the blood vessel wall before extravasation (Figure 2). Interestingly, we have shown that inhibition of McTNs dramatically reduces metastasis [90], indicating that the molecular mechanisms that promote McTNs could serve as a potential therapeutic target to reduce metastasis.

## 4. Promoting Effect of ROS in Metastasis

Cancer cells exhibit an elevated metabolism to primarily support rapid proliferation, biomass production, and survival in nutrient-poor, hypoxic tumor environments [91]. Their elevated metabolism promotes ROS production through multiple mechanisms due to their reprogramming. Acute-to-moderate hypoxia also led to an increase in ROS due to a redox imbalance and back-up of electrons in the electron transport chain [92,93,94]. Beyond initiating DNA damage and primary tumor cell proliferation, oxidative stress and elevated ROS levels can enhance multiple steps within the metastatic cascade. Here, we discuss how NOX activation and hypoxia can induce an EMT and how ROS can alter cell signaling to promote tumor cell migration and invasion during the processes of intra- and extravasation, and we discuss some external sources of ROS from the tumor microenvironment encountered by metastasizing tumor cells.

### 4.1. NOX Activation and EMT

**Figure 4:**
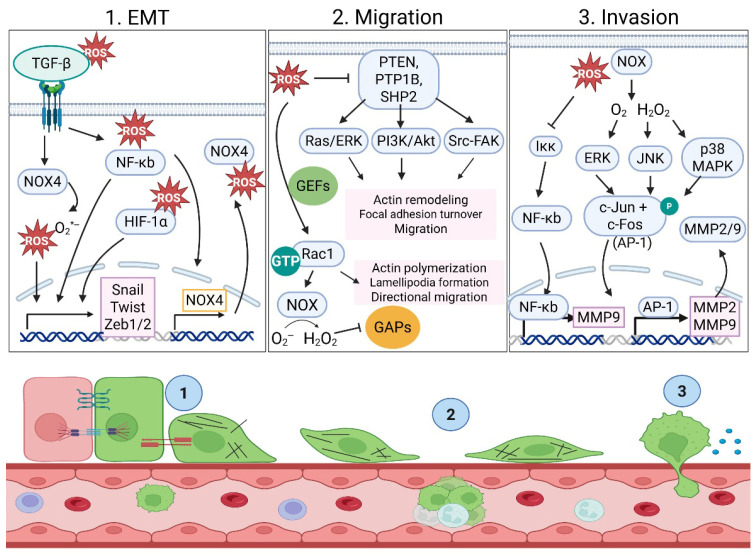
ROS promote EMT, cell migration and cell invasion via multiple mechanisms. Cell signaling pathways stimulated by ROS to promote EMT (1), cell migration (2), and cell invasion (3). As the tumor cells undergo an EMT (pink to green), their morphology changes and they become less adherent to neighboring cells. During an EMT (1), the tumor cells acquire enhanced migratory (2) and invasive capabilities (3) for movement away from the primary tumor and intravasation into the bloodstream. ROS can directly and indirectly regulate TGF-β, NF-κB, and HIF-1α. ROS directly regulate TGF-β by activating its latent form or indirectly by inducing its mRNA and protein expression via signaling pathways such as NF-κB. Directly, nuclear ROS can modify NF-κB subunits and reduce their ability to bind DNA, or ROS can indirectly activate the NF-κB pathway by upstream IKK complex oxidation or via phosphatase inhibition, resulting in prolonged pathway activation. ROS directly regulate HIF-1α via inhibition of PHD activity or via post-translational modification that prevents HIF-1α degradation. ROS can also indirectly regulate HIF-1α via activation of signaling pathways such as PI3K/Akt and MAPK/ERK or via NOX enzymes in a positive feedback loop. ROS directly regulate PTPs (PTEN, PTP1B, SHP2) via active-site cysteine oxidation. NOX4 is considered constitutively active, so it produces ROS without stimuli. NOX4-derived ROS can lead to increased mitochondrial ROS and activate signaling pathways, such as PI3K/AKT or NF-κB, which, in turn, can further upregulate NOX4 expression, creating a self-perpetuating cycle. Created in BioRender. Ju, J.A. (2026) https://BioRender.com/c2448457 (accessed on 25 March 2026).

ROS promote epithelial-to-mesenchymal transition (EMT) through several interconnected mechanisms and signaling pathways. The most described mechanisms involve activation of transcription factors and modulation of key cellular pathways. ROS upregulate EMT transcription factors such as Snail, Twist, and ZEB1/2, particularly via NF-κB-dependent signaling [95,96]. Under normoxic conditions (21% O_2_), NOX1 activation produces H_2_O_2_ to promote a Snail-induced EMT via NF-κB pathway activation, which can be blocked by the ROS scavenger N-acetyl-cysteine (NAC) [97,98]. In A549 lung cancer cells, NF-κB transcriptionally drove NOX4 expression in response to TGF-β. NOX4 upregulation increased ROS to promote a Snail-induced EMT. Either an NF-κB inhibitor or NOX inhibitor was able to inhibit the EMT [99]. In glioblastoma, however, the TGF-β-induced NOX4 is mediated via SMAD3 to induce metabolic reprogramming. While an EMT shift was determined by an increase in the mesenchymal markers N-cadherin and vimentin and phenotypic behaviors such as migration and invasion, the status of any EMT transcription factor was not examined [100]. In both nontumorigenic mammary epithelial MCF-10A cells and metastatic triple-negative breast cancer MDA-MB-231 cells, TGF-β treatment upregulated NOX4 and the production of subsequent extracellular superoxide to induce an EMT (Figure 4) [101,102]. Thus, ROS production via NOX expression and/or activation can drive an EMT shift.

### 4.2. Hypoxia and EMT

Hypoxia-induced oxidative stress is mainly due to increasing mitochondrial and non-mitochondrial ROS production while simultaneously impairing antioxidant defenses. ROS generated from acute-to-moderate hypoxia leads to the stabilization of Hypoxia-Inducible Factor 1 alpha (HIF-1α) by inhibiting prolyl hydroxylases (PHDs), which mark HIF-1α for proteasomal degradation [103]. As HIF-1α levels decline and the cellular response shifts from acute adaptation to chronic hypoxia management, HIF-2α and/or HIF-3α may increase. While HIF-1α is the best-characterized HIF isoform in EMT, there is some evidence to suggest that HIF-2α and HIF-3α may complement HIF-1α in promoting an EMT [104,105,106]. However, HIF-1α is considered the master regulator protein that controls the expression of genes involved in an acute hypoxic response and directly binds to hypoxia response elements (HRE, 5′-RCGTG-3′) in the promoters of *Twist*, *Snail* and *ZEB1* [107,108]. *Twist* and *Snail* are most robustly transcriptionally upregulated by HIF-1α in epithelial and cancer cells, but *ZEB1* also contains HRE sites for HIF-1α binding (Figure 4) [108,109,110,111]. HIF-1α does not directly bind to the *Slug* or *ZEB2* promoters. Instead, these EMT-driving transcription factors are indirectly induced via NF-κB and Twist networks [112]. While hypoxia acts as a powerful trigger for oxidative stress, creating conditions where ROS accumulate despite low oxygen levels, hypoxia can also induce an EMT via transcriptional activation of EMT-promoting factors.

### 4.3. Intravasation and Extravasation

#### 4.3.1. Tumor Cell Migration

ROS promote tumor cell migration through multiple interconnected mechanisms that remodel the cytoskeleton, alter adhesion dynamics, and drive pro-migratory gene expression. ROS can amplify kinase signaling by inactivating key phosphatases controlling the intensity or duration of cellular signals. The best-studied mechanism involves H_2_O_2_ generated from superoxide (O_2_*^−^) produced by NOX or mitochondria to oxidize phosphatases [113]. By targeting a catalytic cysteine residue in active sites of specific phosphatases, through a series of reversible or irreversible modifications, ROS inactivate these enzymes. Examples of ROS-targeted phosphatases are PTEN, PTP1B, SHP2, and LMW-PTPs (Figure 4) [114,115,116,117,118]. Their inactivation leads to overactive PI3K/AKT, Ras/ERK, and/or Src-focal adhesion kinase (FAK) signaling, thus enhancing actin remodeling, focal adhesion turnover, and tumor cell migration [119,120]. In addition to phosphatase inactivation, ROS can activate Rac1 and other Rho-family GTPases to coordinate actin polymerization, lamellipodia formation, and directional migration by enhancing guanine exchange factor (GEF) activity or inhibition relief, thereby increasing Rac1-GTP levels [121]. In cancer cells, growth factor or integrin signaling generates NOX-derived ROS to amplify Rac1 activation by stabilizing or recruiting GEFs to the plasma membrane, promoting lamellipodial protrusion [122]. Rac1-GTP can bind NOX subunits directly, activating NOX complexes and generating superoxide, which is rapidly converted to H_2_O_2_. This NOX-derived H_2_O_2_ can oxidize and inhibit Rac1-GTPase-activating proteins (GAPs) or phosphatases that would otherwise terminate Rac1 activity. Thus, Rac1 and ROS can engage in a positive feedback loop where Rac1 stimulates ROS production and ROS, in turn, sustain Rac1 activation [123]. Inhibiting these negative regulators (Rac1-GAPs or phosphatases) shifts the balance toward Rac1-dependent actin polymerization at the leading edge, supporting persistent lamellipodia and forward movement [121,124].

Alternatively, an increase in or chronic ROS contributes to aberrant cell migration by modulating cytoskeletal proteins and dynamics [125,126,127,128,129]. NOX-generated ROS can promote actin polymerization in the front of the cell by targeting specific cysteine residues (Cys139 and Cys147) in cofilin, forming intramolecular disulfide bonds that inactivate its actin-severing activity, promoting actin polymerization [127,128]. However, ROS have also been shown to indirectly promote cofilin binding to F-actin by oxidizing 14-3-3, which allows the cofilin phosphatase SSH-1L to be released and dephosphorylate cofilin [126]. LIM-Kinase (LIMK) is responsible for phosphorylation and cofilin inactivation. While ROS do not typically target LIMK directly, they tune LIMK activity by modulating upstream GTPases, kinases, and phosphatases in the Rho/ROCK–LIMK–cofilin and Rac/Cdc42–PAK–LIMK–cofilin pathways. ROS are also necessary for the establishment of cell polarity during PDGF-stimulated directional migration. Depleting ROS by NOX1 removal disrupts the phosphatase PP2A, leading to aberrant phosphorylation and activation of the Par3/aPKC/Tiam polarity complex, which promotes multiple small lamellipodia-like protrusions from the periphery of the cell instead of the appropriate formation of a single, polarized lamellipodia. The re-expression of NOX1 or exogenous H_2_O_2_ reverses this phenotype [130]. Thus NOX1-induced ROS can control aPKC and Par3 phosphorylation by regulating PP2A activity, leading to unique and functional lamellipodia. Another study shows that superoxide production by NOX5 is stimulated by actin effector molecules, and knockdown of NOX5 impairs pancreatic cancer cell migration [131]. L-plastin (LPL) is an actin-bundling protein that, when oxidized via H_2_O_2_, has diminished actin-bundling capacity, leading to inhibited tumor cell spreading and reduced migration [129]. While multiple cysteines in actin have been reported to undergo oxidation, there is not yet an example of localized oxidation of actin during cell migration [125]. These studies begin to elucidate how ROS affect multiple points in signaling pathways to control actin dynamics and polarity to affect cell migration.

ROS can also alter adhesion dynamics by modulating cell–cell junctions, integrin signaling, and focal adhesions through redox-sensitive kinases, phosphatases, and cytoskeletal regulators. One mechanism by which ROS mediates the weakening of the cell–cell junctions is by inhibiting PTPs responsible for dephosphorylating b-catenin. Phosphorylation of b-catenin disrupts association with E-cadherin, indicating that ROS can help convert junctional disengagement into a pro-migratory program [132,133]. In head-and-neck squamous cell carcinoma (HSNCC), loss of E-cadherin-mediated cell adhesion and cell–cell interactions triggered Rac1–NOX–ROS, which activated the nonreceptor tyrosine kinase Src and STAT3 to drive migration [134]. Integrins connect the extracellular matrix (ECM) to the intracellular cytoskeleton and are the point of internal focal adhesion (FA) complex assembly. ROS can enhance integrin clustering and activation and modify FA components, influencing their dynamics and impacting cell adhesion, migration, and downstream signal transduction [135]. Multiple redox-sensitive pathways tune the lifetime and size of FA, favoring smaller, more dynamic adhesions that support faster, directional migration [136]. In addition to integrin clustering, ROS can oxidize regulatory cysteines in Src, with Cys245 in the SH2 domain and Cys487 in the kinase domain being the main redox-sensitive residues [137]. Oxidation favors a conformational change, opening the protein for autophosphorylation of Tyr416 in the activation loop to then activating FAK, which enhances focal adhesion turnover and actin remodeling to promote cell migration. As previously mentioned, ROS can also reversibly oxidize cysteine residues in PTPs that normally dephosphorylate Src and FAK, prolonging their activity [118]. Sustained Src–FAK signaling increases phosphorylation of paxillin and talin, promoting FA turnover at the leading edge, which is required for persistent migration [117,135]. Supporting the importance of Src and FAK in metastasis is the fact that an increased Src or FAK expression or activity has been identified in many types of cancer and is correlated with a poor prognosis [138,139,140,141]. These studies identify the importance of ROS in remodeling the cytoskeleton and in altering adhesion dynamics to induce a pro-migratory phenotype.

#### 4.3.2. Tumor Cell Invasion: ROS Regulation of MMPs

Matrix metalloproteinases (MMPs) are a family of proteolytic enzymes that degrade components of the extracellular matrix to facilitate cancer cell invasion, with MMP2 and MMP9 (gelatinases) particularly implicated in cancer spread. There are multiple examples of ROS promoting cancer cell invasion via MMP2/MMP9 expression or activity, while antioxidants decrease MMP2-/MMP9-stimulated invasion [142,143,144,145,146,147,148]. ROS, particularly H_2_O_2_ and mitochondrial/NOX-derived superoxide, activate the ERK, JNK, and p38 MAPK pathways, leading to phosphorylation of c-Jun and c-Fos and formation of the AP-1 complex, which binds to the promoters of *MMP2* and *MMP9* to increase their mRNA and protein expression (Figure 4) [149,150,151,152,153,154,155]. Although *MMP2* is often described as less dependent on AP-1 than *MMP9*, perhaps in part because it is basally active, functional AP-1 sites exist within its promoter and contribute to its transcription in several cell types [156,157]. ROS can upregulate *MMP2* and *MMP9* via other transcription factors, including ETS factors, HIF-1α, NF-κB, or ATF-2 [158,159,160,161,162,163]. HIF-1α, stabilized by ROS, can directly transactivate the *MMP9* promoter or indirectly transactivate the *MMP2* promoter via additional factors or intermediate regulators to increase mRNA and protein [164,165,166]. NF-κB can be activated by ROS oxidizing and inactivating IκB or by modulating upstream kinases such as IKK, leading to NF-κB nuclear translocation and MMP-9 upregulation [167,168]. ROS can also stabilize MMP9 mRNA. Mori et al. determined that targeted reduction of hydrogen peroxide-inducible clone-5 (HIC-5) promoted lung metastasis in mice after tail vein injection without affecting primary tumor growth. The mechanism for enhanced metastasis followed a HIC-5-NOX4-mtROS-MMP9 axis [169]. In addition to ROS promoting transcriptional upregulation of MMP2 and/or MMP9 or stabilizing MMP9 mRNA, ROS can post-translationally activate MMP2 and MMP9. MMPs are latent proproteins held in an inactive conformation by the cysteine in the prodomain interacting with Zn^2+^ bound to the catalytic domain. Hydrogen peroxide, peroxynitrate, nitric oxide and the hydroxyl radical can oxidize the cysteine thiol in the prodomain to disrupt its interaction with Zn^2+.^, causing a “cysteine switch” and enzyme activation [170]. The versatility of ROS in activating MMPs at multiple levels underscores their role in metastasis.

### 4.4. CTC Survival and Immune Cell Evasion

**Figure 5:**
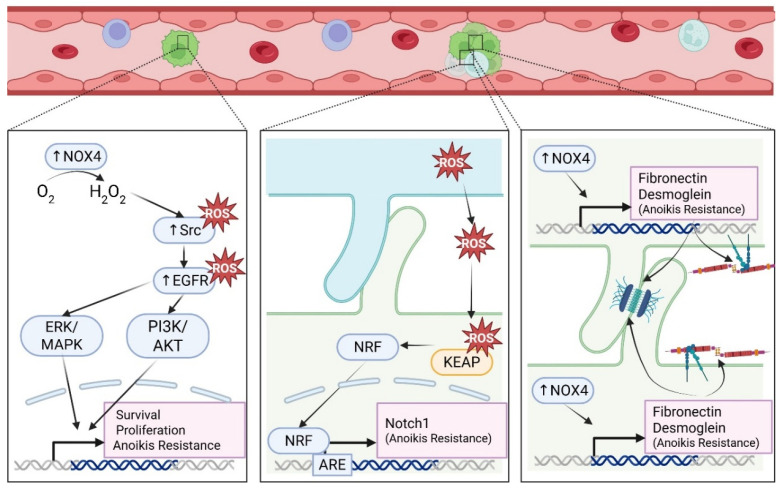
ROS can aid in CTC survival. Upregulation of NOX4 (vertical arrow pointing up) can enhance anoikis resistance and CTC survival via upregulation of the MAPK and PI3K pathways. NOX4-derived ROS can inhibit Src activity via direct oxidation of a cysteine residue, acting as a “redox” switch to limit Src activity during high oxidative stress, and ROS can also indirectly activate Src by inhibiting PTPs that normally turn it off. For EGFR, ROS can directly oxidize cysteine in EGFR’s kinase domain to enhance tyrosine kinase activity and indirectly activate signaling intermediary proteins by inactivating negative regulators such as PTPs, driving EGFR phosphorylation and downstream signaling. Peripheral mononuclear-myeloid-derived suppressor cells (PMN-MDSCs) and tumor cells form heterotypic clusters. The ROS generated from PMN-MDSCs, primarily from high activity of NOX2, act on associated CTCs to directly inhibit KEAP1 binding to NRF2 and activate the NRF2-ARE axis to induce Notch1 gene expression and promote anoikis resistance. Homotypic cell clustering induces a NOX4-mediated upregulation in fibronectin and desmosomal proteins to promote cancer cell aggregation and facilitate anoikis resistance. Created in BioRender. Gilchrist, D.E. (2026) https://BioRender.com/c2448457 (accessed on 25 March 2026).

The most important parameter that determines the successful metastasis of tumor cells is CTC survival [171]. One of the survival challenges CTCs must overcome is apoptotic cell death caused by ECM detachment, also known as anoikis. When epithelial-originating tumor cells detach from ECM, they experience a loss in pro-survival signaling pathways, resulting in disinhibition of the apoptotic pathways. Cancer cells have developed abrogated metabolic mechanisms that alter redox balance to circumvent cell death pathways and ultimately become resistant to anoikis [172]. NOX enzymes have been identified as contributors to ROS-dependent anoikis resistance. NOX4 was identified as an oncogene that is overexpressed in tumorigenic breast cell lines, primary breast tumors, and primary ovarian tumors [173]. NOX4-overexpressing MCF-10A cells demonstrated resistance to Etoposide-induced apoptosis, in addition to induction of other tumorigenic phenotypes, including anchorage-independent growth, invasion and cell division. In gastric and lung cancer cells, NOX4 is upregulated in suspended cells relative to attached cells and confers anoikis resistance [174,175]. NOX4-mediated anoikis resistance occurred via elevation of ROS levels, leading to oxidation and activation of Src and EGFR, which sustained suspended cell survival through the activation of the PI3K/AKT and ERK/MAPK signaling pathways (Figure 5) [174,175]. Furthermore, NOX4 overexpression and ROS treatment increased EGFR levels to promote anoikis resistance, which was attenuated upon NOX4 knockdown and depletion of ROS. An in vivo xenograft colorectal cancer (CRC) model revealed NOX4 inhibition reduced tumor growth and lung metastasis [176]. Although this study did not investigate suspended CRC cells, it is clear that NOX4 plays an essential role in regulation of apoptotic cell death in multiple solid tumors [177]. The overexpression of NOX4 in both CRC and oral tongue squamous cell carcinoma was shown to be associated with poor overall survival [176,178]. Beyond the tumor-intrinsic contributions of NOX4-generated ROS, tumor-extrinsic NOX4 expression in cancer-associated fibroblasts exert tumor-promoting functions [179]. To demonstrate the effect of NOX4 in the stroma, mouse mammary tumor cells were implanted into wild-type mice and mice with a constitutive deletion of NOX4 (*Nox4^-/-^*). The mice with *Nox4* deleted had a reduced tumor volume and a significant decrease in distant metastases compared to the wild-type control mice, suggesting that the oxidative tumor microenvironment promotes tumor cell survival via paracrine signaling pathways. Overexpression of NOX4 is associated with poor patient survival, and its inhibition reduces tumor growth and metastasis, identifying it as a key therapeutic target.

Isolation of CTC clusters from breast cancer patients revealed the majority (85.5–91.7%) of CTC-associated white blood cells to be Ly-6G positive cells with neutrophil nuclear morphology, suggesting CTC–neutrophil clusters to be the primary CTC–cell interaction in circulation aside from other tumor cells [180]. We have recently shown that, in addition to cancer cells, neutrophil-differentiated HL-60 cells and primary human neutrophils form McTNs to aid in heterotypic tumor cell clustering [181]. Circulating granulocytic peripheral mononuclear-myeloid-derived suppressor cells (PMN-MDSCs) are often referred to as “pathologically activated neutrophils” and have been found in heterotypic clusters with CTCs isolated from melanoma and breast cancer patients. When a combination of patient-isolated PMN-MDSCs and brain metastatic breast cancer cells (MDA-MB-231BR) were introduced into mice via intracardiac injection, the combination enhanced tumor cell dissemination [182]. PMN-MDSCs facilitated CTC survival through ROS generation, which activates the NRF2-ARE axis and induces Notch1 gene expression in the associated CTCs (Figure 5). Suspended lung and breast cancer cell lines induce NOX4-mediated upregulation in fibronectin and desmosomal proteins to facilitate anoikis resistance through cell aggregate formation (Figure 5) [183]. It is possible that McTN-mediated cell clustering brings tumor cells within close proximity to peripheral blood immune cells that can support this ROS-dependent CTC survival.

### 4.5. Redox-Dependent Metabolic Reprogramming: DTC Dormancy

Driven by stresses at the secondary site, DTCs can enter into dormancy, a state of reversible cell cycle arrest, as a protective survival mechanism to avoid apoptosis. The new microenvironment is typically one of high oxidative stress. Additionally, ROS can damage components of the electron transport chain and inhibit ROS-sensitive enzymes like aconitase, both reducing the forward (oxidative) flux of the TCA cycle and making oxidative metabolism less efficient. Pushing the cells to metabolically adapt, the cells shift away from glycolysis towards mitochondrial oxidative phosphorylation (OXPHOS) and fatty acid oxidation [184]. Nuclear factor erythroid 2-related factor 2 (NRF2) is known to be a master regulator in response to oxidative stress [185], and multiple studies have identified NRF2 target genes that regulate glycolysis, glycogen metabolism, one-carbon metabolism, nucleotide metabolism, fatty acid metabolism, glutamine metabolism, the pentose phosphate pathway and glutathione metabolism [186,187]. While studying metabolic changes in dormant breast cancer cells, Fox et al. determined that the dormant cells upregulated NRF2 to manage increased ROS cells and indeed induced an NRF2 antioxidant transcriptional program [187]. In the absence of oxidative stress, NRF2 is bound by Kelch-like ECH-associated protein 1 (KEAP1) and targeted for degradation, but ROS can oxidize critical cysteine residues on KEAP1 to induce a conformational change, preventing NRF2 binding. In the absence of KEAP1 binding, NRF2 can then accumulate, translocate into the nucleus, and bind to antioxidant elements (AREs) in gene promoters [188]. High NRF2 levels not only sustain dormancy, but direct manipulation of NRF2 activity was shown to also facilitate the metabolic reprogramming required for dormant breast cancer cells to start growing again. However, persistant ROS were determined not to be the cause of the elevated levels of NRF2 in the recurrent tumors. Instead, NRF2 elevation was possibly due to a noncanonical mechanism such as impaired protein degradation [187].

As an alternative to glutamine metabolism and a way to maintain biosynthesis, dormant cells can switch to reductive carboxylation, which is basically a reversal of a part of the TCA cycle. During reductive carboxylation, α-ketoglutarate, a primary glutamine metabolite, is converted to isocitrate and then citrate using NADPH. The produced citrate is transported into the mitochondria to detoxify ROS via glutathione and related antioxidant systems. Reductive carboxylation typically increases under conditions of low oxygen; however, cells grown as anchorage-independent spheroids increase reductive carboxylation to detoxify ROS under normal oxygen levels [189]. This suppression of conventional metabolism and induced reductive carboxylation was determined to be dependent on isocitrate dehydrogenase 1 (IDH1). Depending on the type of cancer, DTCs can persist in a dormant state for many years until being “reawakened” to proliferate by appropriate stimuli [190].

## 5. Challenges/Controversies/Remaining Gaps

### 5.1. The ROS Seesaw Effect

Thus far, we have highlighted the metastasis-promoting roles of ROS; however, the sensitive redox homeostasis of a tumor cell can tip the cell from pro-metastatic to anti-metastatic. High ROS levels can be detrimental to the survival of cancer cells by overwhelming their upregulated antioxidant systems and damaging essential proteins and nucleic acids via oxidation. Upon loss of matrix attachment, MCF-10A breast epithelial cells experienced reduced cellular ATP levels and elevated ROS levels [191]. NAC and Trolox antioxidants eliminated detachment-induced ROS and rescued cellular ATP levels to support anchorage-independent cell survival and luminal filling of cells during MCF-10A acini formation. Thus, cellular adaptation to remove excess ROS and reduce oxidative stress could actually support CTC survival. This was further validated by the expression or knockdown of catalase enzyme to demonstrate its critical role to support anchorage-independent growth in MCF-10A, MDA-MB-231, and T47D breast cells [192]. Amongst a screen of small molecules that could be used for successful ex vivo expansion of single CTCs isolated from breast cancer patients, NAC proved to be the best compound across four primary CTC lines. NAC treatment was sufficient to rescue CTC proliferation, likely through metabolic alterations that mitigate oxidative stress [193].

While low or moderate ROS levels can act as signaling messengers that can promote cell movement, high levels cause excessive protein oxidation, DNA damage, and cell death, overriding pro-migratory and pro-invasion pathways. Extremely high levels of ROS induce mitochondrial dysfunction, leading to both caspase-dependent and -independent apoptosis [31]. As mentioned, ROS can promote actin polymerization to enhance migration, but levels that are not spatially regulated and/or too high can restrict the cell’s ability to move by altering its mechanical properties [194]. High ROS can trigger F-actin polymerization specifically at the actin cortex, which makes the cell more rigid, significantly slowing migration rates, an effect that can be reversed by NAC treatment. Amino acids within microtubules and actin are susceptible to oxidation, and thus excess ROS can interfere with cell movement by direct oxidation of the cytoskeleton. High ROS can cause actin filament severing and reduced microtubule polymerization, effectively freezing the cell’s internal transport and structural remodeling. As mentioned, spatially controlled NOX-induced ROS can oxidize and inactivate cofilin to promote actin polymerization at the leading edge of the cell. However, excess ROS can lead to uncontrolled cofilin inactivation, where, although cofilin may be able to bind actin, it loses its ability to sever or depolymerize the filaments, completely halting actin turnover [195]. Additionally, ROS can deplete energy that is essential for migration by activating poly (ADP-ribose) polymerase (PARP-1) upon DNA damage, which consumes NAD+ and inhibits glycolysis, leading to ATP depletion [196]. Excessive ROS can also inhibit PTPs by irreversible oxidation rather than reversible signaling, altering the phosphorylation balance necessary for focal adhesion turnover.

### 5.2. Novel ROS Detection Approaches

**Figure 6:**
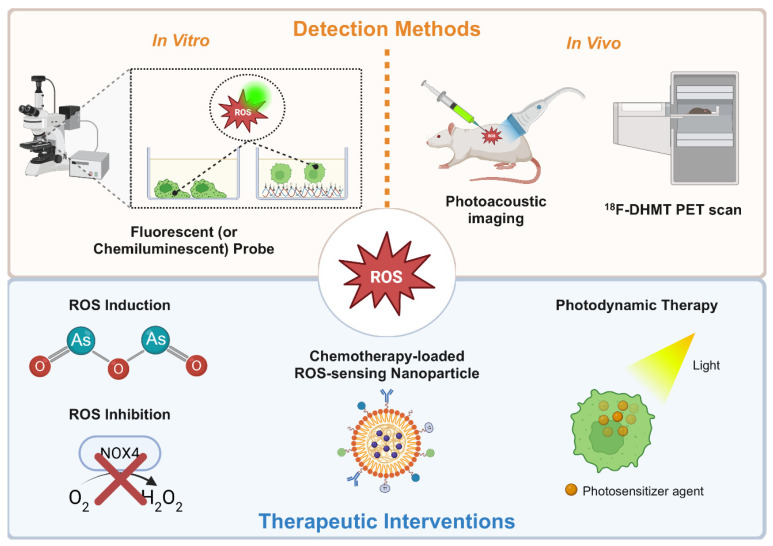
ROS detection methods and therapeutic interventions. In vitro and in vivo ROS detection methods and ROS-centered therapeutic strategies aiming to alter metabolic state of tumor cells by manipulating ROS levels. Created in BioRender. Glichrist, D.E. (2026) https://BioRender.com/c2448457 (accessed on 25 March 2026).

ROS play a dynamic pathophysiological role throughout the metastatic cascade that is determined by the levels of ROS (high or low), the source of ROS and the stage of metastasis (EMT, invasion, migration, intravasation/extravasation, and circulation). As a result, biomolecular markers for real-time detection methods to assess ROS activity in various stages of the metastatic cascade are critical to understand the metastatic propensity of tumor cells. The rapid redox fluctuations, short half-lives, and high reactivity of ROS enhance the difficulty of their direct, real-time measurement. However, novel approaches to measure ROS in vitro and in vivo are continually being developed. Most in vitro methods to measure ROS are commercial kits with fluorescent or chemiluminescent probes that interact with all ROS species or are species-selective [197]. Oxidation products have also been used as a measure of cellular ROS activity. Integration of these probes with poly-HEMA-coated tissue culture plates enabled the assessment of ROS roles in extracellular matrix-detached cancer cells [198]. DCF-DA, CellROX, and MitoSOX fluorescent ROS indicators were applied to detached cells to elucidate pro-survival roles of antioxidant activity in suspended cells, representative of circulating tumor cells (Figure 6) [191]. Our group patented a novel optically clear cell tethering nanosurface technology, TetherChip, to spatially immobilize nonadherent cells. TetherChip technology engages hydrophobic interactions with the cell plasma membrane to spatially immobilize cells while simultaneously preventing the formation of protein-based adhesions [79]. TetherChip has enabled the elucidation of key biomolecular mechanisms, including tubulin-driven protrusions (McTNs) that support metastatic phenotypes in nonadherent cells, which were identified with intracellular immunofluorescence approaches [79,81,82,83,84,85,86,87,88,89,90,181,199,200,201,202,203,204]. It is possible TetherChip technology can be incorporated with ROS detection probes in live tumor cells to advance our understanding of the pro-survival or pro-death roles of ROS in matrix-detached states.

Beyond these effective in vitro ROS detection methods, in vivo detection and quantification of ROS will enable minimally invasive, real-time assessment of patient tumors. A novel photoacoustic and fluorescent probe, JW41, was developed for H_2_O_2_-selective detection and quantification, proving to be effective in vitro and in vivo [205]. The capped near-infrared probe (JW41) irreversibly reacts with supraphysiological levels of H_2_O_2_, which forms an uncapped derivative (JW35) that experiences a shift in the absorption spectrum to be detected by photoacoustic and fluorescent imaging. This probe was evaluated to be stable in plasma at 37 °C, nontoxic to MCF-7 and MDA-MB-231 breast cancer cells and taken up efficiently via glucose transporters. In vivo testing with JW41 demonstrated specific accumulation and retention in tumor tissue with spectral signals observed from conversion to the JW35 derivative up to 24 h. A chemoselective bioluminescent probe, Peroxy Caged Luciferin-1 (PCL-1), afforded real-time detection of H_2_O_2_ in androgen-sensitive prostate tumor (LNCaP) xenograft mice models [206]. Interaction with increasing concentrations of H_2_O_2_ releases firefly luciferin from PCL-1 to produce increasing total photon flux upon luciferase exposure. This probe was able to detect real-time ROS fluctuations in LNCaP tumors following testosterone stimulation or NAC treatment of the tumors. To detect early signs of doxorubicin-induced cardiotoxicity in cancer patients, a novel positron emission tomography (PET) tracer, ^18^F-DHMT, was used to identify elevated ROS production [207]. ^18^F-DHMT radiotracer was introduced in rats to demonstrate sensitive detection of early increases in myocardial superoxide levels to establish an effective noninvasive detection method. While this work focused on usage in chemotherapy-induced cardiotoxicity, it is possible that ^18^F-DHMT could be applied with the standard of care imaging platform, PET, as a detection method for elevated ROS in primary or circulating tumor cells to indicate EMT, cell survival, and other pro-metastatic phenotypes.

### 5.3. Translational Considerations and Redox-Modulating Agents

Tumor cells take advantage of ROS signaling to exert pro-metastatic phenotypes that contribute to poor patient outcomes. However, we also highlighted that tumor cells can leverage antioxidants to reduce oxidative stress burden, thus maintaining an optimal level of elevated ROS to be pro-tumorigenic without deleterious effects. As a result, current ROS-centered therapeutic strategies aim to disrupt that altered metabolic state of tumor cells by increasing ROS beyond manageable levels or reducing ROS below pro-tumorigenic/metastatic levels (Figure 6). Over the years, multiple ROS-modulating drugs have been tested in clinical trials. Antioxidant inhibitors such as auranofin, buthionine sulfoximine (BSO), PX-12, disulfiram and ATN-224 selectively disable protective antioxidants systems (Table 2). The anti-rheumatic drug auranofin inhibits thioredoxin reductase and promotes the accumulation of ROS by disrupting the thioredoxin antioxidant system and is being repurposed for cancer treatment. Combined auranofin and sirolimus for advanced lung cancer (NCT01737502) was generally tolerable but produced only modest clinical benefit, with a median overall survival of 4.4 months. While this specific combination did not support a new standard of care, auranofin is advancing into other trials combined with immune checkpoint inhibitors, targeting different cancers such as recurrent glioblastoma and ovarian cancer. BSO has been tested in multiple Phase 1 clinical trials but almost exclusively in combination with chemotherapy agents to overcome drug resistance by depleting glutathione in cancer cells [208]. Similarly, PX-12 showed promise when used in combination with chemotherapy to overcome drug resistance, but these combinations largely remain at the research stage rather than progressing into clinical trials. Disulfiram in the presence of copper forms a potent anticancer complex (CuET) that increases intracellular ROS and inhibits the NF-κB pathway and ubiquitin proteosome system. Unfortunately, disulfiram has proven unsuccessful thus far in multiple clinical trials due to poor stability, rapid metabolism, and/or short half-life within the plasma, which reduces the accumulation of necessary CuET concentrations in the tumor microenvironment [209]. Finally, ATN-224 is a copper chelator that leads to the inhibition of the copper-/zinc-dependent enzyme SOD1, increasing intracellular superoxide and decreasing hydrogen peroxide levels, and it has shown limited-to-moderate success as a cancer treatment [210].

Prooxidant agents such as Elesclomol, arsenic trioxide, high-dose vitamin C and artesunate directly generate harmful ROS to promote cell death. Unfortunately, a Phase 3 clinical trial (NCT00522834) using Elesclomol in combination with paclitaxel for treatment of metastatic melanoma was stopped early due to an imbalance in deaths [211]. However, the results from a 2025 completed Phase 3 clinical trial (NCT02688140) supports the use of the FDA-approved arsenic trioxide (ATO, As_2_O_3_) in combination with all-trans retinoic acid (ATRA) for the treatment of acute promyelocytic leukemia (APL) [212]. Intravenous, high-dose vitamin C in combination with chemotherapy and radiation has shown promising results in Phase 2 and 3 clinical trials [213]. For metastatic pancreatic cancer, adding high-dose vitamin C to standard chemotherapy doubled the median overall survival for patients from 8 to 16 months (NCT02905578) [214]. Preliminary findings from Phase 1 (NCT01752491) and Phase 2 (NCT02344355) trials testing the addition of intravenous vitamin C to the treatment regime of glioblastoma patients demonstrated a 5-month increased survival time. Artesunate, primarily used as an antimalarial, is being tested for its ability to promote ferroptosis and apoptosis in cancer cells. Results from an early Phase 1 foundational pilot study showed it to be well tolerated while extending survival times for the patients receiving artesunate compared to the placebo (NCT02353026) [215]. These promising results helped to launch a current larger, multicenter Phase 2 trial to test artesunate as a neoadjuvant treatment for colorectal cancer (NCT07095309). Artesunate is also being evaluated in Phase 2 trials for its safety and effectiveness in preventing the progression of pre-cancerous cervical intra-epithelial neoplasia to invasive cancer (NCT04098744 and NCT07095478). Although artesunate is in testing as a monotherapy, most redox modulators show minimal clinical activity alone and are increasingly investigated for their ability to sensitize tumors to radiation or standard chemotherapy.

As discussed, ROS can serve as promoters of tumor growth and metastasis, and the direct reduction in ROS or inhibition of ROS-generating enzymes may pose as beneficial therapeutic approaches. While NOX enzymes are a plausible anticancer redox target, most evidence testing NOX-targeted treatment are still in the preclinical or early clinical stages. However, Setanaxib (GKT137831) is an oral, first-in-class inhibitor of NOX1/4 and has received the FDA orphan drug designation (ODD) for treatment against systemic sclerosis, idiopathic pulmonary fibrosis, and primary biliary cholangitis (NCT03865927, NCT05014672) [216]. Considering the elucidated role of NOX4 in tumor cell survival that supports metastatic propensity, Setanaxib would be an interesting drug to test against solid tumors. Thus far, a Phase 2 clinical trial combining Setanaxib with Pembrolizumab in patients with recurrent or metastatic squamous cell carcinoma of head and neck cancer showed significant improvements in progression-free survival (PFS) and overall survival (OS) (NCT05323656). A novel approach to overcome resistance to radiotherapy against tumor cells encapsulated Setanaxib into a bioactive and CD44-targeted hyaluronic acid nanoparticle (HANP) [217]. The nanoparticle payload was systemically delivered to breast cancer patient-derived xenograft (PDX) models along with low-dose local radiotherapy and demonstrated tumor growth inhibition and elevated cell death. As of April 2026, the general trend shows a clear split between the described redox modulators being used in specific combinations and those which have stalled due to efficacy and toxicity issues. Studies are shifting away from BSO, PX-12, ATN-224, and Elesclomol for the treatment of cancers, but Auranofin, vitamin C, artesunate, Setanaxib, and APX3330 show promise and are moving forward into specialized or combination trials.

The FDA has also approved photodynamic therapy (PDT) using light-activated photosensitizers to generate ROS and kill cancer cells (Figure 6). PDT has been used to treat multiple pre-cancerous and cancerous solid tumors, including esophageal cancer, non-small-cell lung cancer, and skin cancer [218]. Current clinical trials often focus on intraoperative or endoscopic delivery to target residual cells that surgery might miss. Though beneficial for reducing tumors that exist specifically at the irradiated site, PDT is not as effective at reducing CTCs or disseminated metastases undetected by current imaging modalities. While not an FDA-approved therapy, Wang et al. demonstrated that their docetaxel-loaded pH/ROS dual-responsive nanoparticle shows potential for preventing tumor metastasis. The intracellular acidic environment and endogenous ROS accelerate the degradation of the nanomaterial, and a component from the degraded material induces more ROS through mitochondrial damage to amplify the docetaxel payload release [219]. This nanoparticle demonstrated tumor-selective capabilities due to the agent-release mechanisms and efficacy to reduce in vivo tumor growth and metastasis. Although our group showed that Taxol agents can induce metastatic phenotypes, this nanoparticle platform may serve as a strong candidate to carry anti-tumor and anti-metastatic therapeutic payloads that induce tumor cell death through oxidative stress mechanisms [220]. These novel approaches to challenge tumor cells with reduced ROS or increased ROS to induce cell death along each stage of the metastatic cascade raise excitement for the advancement of cancer therapies.

## 6. Conclusions

In this review, we have highlighted the dynamic, bifunctional nature of reactive oxygen species (ROS) in tumor metastasis. ROS species are produced through redox reactions and ROS-producing enzymes or as byproducts of normal metabolic activity. ROS levels can overwhelm normal cellular antioxidant systems to cause damage and even cell death. On the other hand, elevated ROS in malignant tumor cells can exert pro-metastatic phenotypes in a stage-dependent manner. Hypoxia-induced and NOX-derived ROS facilitate the epithelial–mesenchymal transition to increase cell motility, invasive capacity, and resilience against internal or external stressors. Once tumor cells have entered the vasculature, ROS activate intracellular and paracrine pro-survival, anti-apoptotic signaling mechanisms to support CTCs during the harsh conditions experienced in circulation. Although substantial evidence demonstrates ROS as pro-metastatic factors, we have discussed in this review mechanisms by which ROS can be damaging to cells. Therefore, there is evidence showing that reduced ROS, via antioxidant upregulation, can also promote metastatic phenotypes. The bifunctional and context-dependent nature of ROS presents a need to develop novel detection methods to investigate ROS at different stages in vitro and in vivo with fluorescent probes and medical imaging modalities. Advancements in our understanding of the multifaceted roles of ROS in metastasis, and other pathological diseases, have led to clinical trials and FDA approval of ROS-centered therapeutics, including ROS inducers, inhibitors, and drug delivery agents. Our ongoing efforts are to elucidate the roles of ROS in each stage of metastasis, to develop novel technologies for ROS detection, and to manufacture or repurpose ROS-sensing, -targeting, or -generating agents to reduce metastasis and improve patient outcomes.

## Figures and Tables

**Table 1:** Characteristics of the 3 primary ROS species.

ROS Species	Main Enzymatic/ Chemical Sources	Steady-State Levels and Lifetime (Physiological Conditions)	Dominant Signaling Roles	Dominant Damaging Roles
Superoxide (O_2_*^-^)	NADPH oxidases ETC Xanthine oxidase Uncoupled NOS ER Peroxisomal oxidases	Very low, <1–10 nM ~10^−6^–10^−5^ s and compartment-restricted	Cytosol: very local redox modulation of enzymes Mitochondria: initiator of mtROS cascades; precursor to mitochondrial H_2_O_2_ that tunes hypoxia signaling, metabolism, and apoptosis Extracellular: NOX-derived superoxide in immune and vascular cells regulates receptor signaling and inflammatory responses	Cytosol/mitochondria: precursor of H_2_O_2_ and ONOO^-^ driving oxidative/nitrosative damage to proteins, lipids, and DNA Extracellular: participates in LDL oxidation and matrix injury via conversion to secondary oxidants
Hydrogen peroxide (H_2_O_2_)	Dismutation of superoxide by SOD1/2/3 Oxidase and dehydrogenase side reactions Peroxisomal oxidases	Low nanomolar to sub-micromolar, often 10–100 nM ~10^−5^–10^−3^ s locally	Cytosol: diffusible second messenger oxidizing Cys in phosphatases and kinases Mitochondria: mtH_2_O_2_ tunes metabolic flux, stress responses, and mitophagy; acts as exported signal to cytosol and nucleus Extracellular: modulates growth factor and cytokine receptor signaling, leukocyte recruitment, and wound responses	Cytosol/nucleus: sustained H_2_O_2_ causes DNA base oxidation and strand breaks, redox enzyme inactivation, and protein carbonylation Mitochondria: promotes mitochondrial protein and lipid oxidation, loss of membrane potential, and release of apoptogenic factors Extracellular: contributes to oxidation of matrix proteins and lipids, promoting vascular and tissue remodeling
Hydroxyl radical (OH*)	Fenton and Haber–Weiss reactions from H_2_O_2_	No measurable steady-state pool, better thought of as local flux rather than measurable uniform value. ~10^−10^–10^−9^ s (essentially instantaneous and nondiffusible)	No meaningful physiological signaling role; damage- dominated	DNA: base modifications, abasic sites, single- and double-strand breaks Proteins: side-chain oxidation, fragmentation, crosslinking, loss of enzymatic activity Lipids: lipid peroxidation in membranes, generating reactive aldehydes

**Table 2:** Redox-modulating drugs tested as cancer treatments.

Drug Category	Drug Name	Target Enzyme/Mechanism	Cancer Type
Antioxidant Inhibitors	Auranofin	Thioredoxin Reductase (TrxR)	Lung Cancer (NSCLC and SCLC) Ovarian Cancer, Chronic Lymphocytic Leukemia (CLL)
	Buthionine Sulfoximine (BSO)	γ-Glutamylcysteine ligase (GCL)	Neuroblastoma, Melanoma, Advanced Solid Tumors
	PX-12	Thioredoxin-1 (Trx-1)	Advanced or Metastatic Cancer
	Disulfiram	ALDH; GSH/GSSG balance	NSCLC, Prostate Cancer, Metastatic Melanoma, Metastatic Breast Cancer
	ATN-224	Superoxide Dismutase 1 (SOD1)	Prostate Cancer, NSCLC, Breast Cancer, Heptocellular Carcinoma, Hematological Malignancies
Prooxidant Agents	Elesclomol	Mitochondrial ROS generation	Metastatic Melanoma
	Arsenic Trioxide	Mitochondrial ROS/TrxR	Acute Promyelocytic Leukemia
	High-dose Vitamin C	Hydrogen Peroxide	Pancreatic Cancer, Glioblastoma, NSCLC, Ovarian and Colorectal Cancers
	Artesunate	Iron-mediated ROS generation	Colorectal and Pre-cancerous Cervical Neoplasia
Redox Catalysts	Setanaxib (GKT137831)	NOX1/NOX4 Inhibitor	Head and Neck Cancer
	APX3330 (E3330)	APE/Ref-1 redox signaling	Advanced Solid Tumors

## Data Availability

No new data were created or analyzed in this study. Data sharing is not applicable to the article.

## Supplementary Materials

The following supporting information can be downloaded at: https://www.mdpi.com/article/10.3390/antiox15050529/s1, Table S1: Characteristics of Additional ROS Species.

## Abbreviations

The following abbreviations are used in this manuscript:

^18^F-DHMT	^18^F-6-(4-((1-(2-fluoroethyl)-1H-1,2,3-triazol-4-yl)methoxy)phenyl)-5-methyl-5,6-dihydrophenanthridine-3,8-diamine
aPKC	Atypical protein kinase C
ALDH	Aldehyde dehydrogenase
APE	Apurinic/apyrimidinic endonuclease 1
APL	Acute promyelocytic leukemia
ARE	Antioxidant elements
ATF2	Activating transcription factor 2
ATO	Arsenic trioxide
ATP	Adenosine triphosphate
ATRA	All-trans retinoic acid
CAT	Catalases
CellROX	Cellular reactive oxygen species indicator probe
c-Fos	Cellular FBJ murine osteogenic sarcoma virus
cGMP	Cyclic guanosine monophosphate
c-Jun	Cellular Jun protein
CLL	Chronic lymphocytic leukemia
CRC	Colorectal cancer
CTC	Circulating tumor cells
DAPI	4′,6-diamidino-2-phenylindole
DCF-DA	2′,7′-dichlorodihydrofluorescein diacetate
DNA	Deoxyribonucleic acid
DTC	Disseminating/disseminated tumor cell
DUOX	Dual oxidase
ECM	Extracellular matrix
EGFR	Epidermal growth factor receptor
EMT	Epithelial-to-mesenchymal transition
eNOS	Endothelial nitric oxide synthase
ER	Endoplasmic reticulum
ERK	Extracellular signal-regulated kinase
ETC	Electron transport chain
ETS	Erythroblast transformation-specific
FA	Focal adhesion
FAD	Flavin adenine dinucleotide
FAK	Focal adhesion kinase
Fas	FS-7-associated surface antigen
FDA	Food and Drug Administration
GAP	GTPase-activating protein
GCL	γ-Glutamylcysteine ligase
GEF	Guanine nucleotide exchange factor
GFP	Green fluorescent protein
GPx	Glutathione peroxidases
GSH	Reduced glutathione
GSSG	Oxidized glutathione
GTP	Guanosine triphosphate
HANP	Hyaluronic acid nanoparticle
HEMA	2-Hydroxyethyl methacrylate
HIC-5	Hydrogen peroxide-inducible clone-5
HIF-1α	Hypoxia-inducible factor 1 alpha
HRE	Hypoxia response elements
HSNCC	Head-and-neck squamous cell carcinoma
IDH1	isocitrate dehydrogenase 1
IKK	Inhibitor of kappa beta kinase
iNOS	inducible nitric oxide synthase
JNK	c-Jun N-terminal kinase
KEAP1	Kelch-like ECH-associated protein 1
KGDHC	α-Ketoglutarate dehydrogenase complex
LDL	Low-density lipoprotein
LIMK	LIM domain kinase
LMW-PTP	Low-molecular-weight protein tyrosine phosphatase
LPL	L-plastin
Ly-6G	Lymphocyte antigen 6 complex, locus G
MAPK	Mitogen-activated protein kinase
McTNs	Microtentacles
MitoSOX	Mitochondrial superoxide indicator
MMPs	Matrix metalloproteinases
mRNA	Messenger ribonucleic acid
mtROS	Mitochondrial reactive oxygen species
NAC	N-acetylcysteine
NADH	Nicotinamide adenine dinucleotide
NADPH	Nicotinamide adenine dinucleotide phosphate
NCT########	National clinical trial (registered on ClinicalTrials.gov) [221]
NF-κB	Nuclear factor kappa-light-chain-enhancer of activated B cells
nNOS	Neuronal nitric oxide synthase
NOS	Nitric oxide synthase
NOX	NADPH oxidases
NRF2	Nuclear factor erythroid 2-related factor 2
NSCLC	Non-small-cell lung cancer
ODD	Orphan drug designation
OS	Overall survival
OXPHOS	Oxidative phosphorylation
PAK	p21-activated kinases
Par3	Partitioning-defective 3
PARP	Poly (ADP-ribose) polymerase
PCL-1	Peroxy caged luciferin-1
PDGF	Platelet-derived growth factor
PDT	Photodynamic therapy
PET	Positron emission tomography
PFS	Progression-free survival
PHD	Prolyl hydroxylase
PI3K	Phosphoinositide 3-kinase
PMN-MDSC	Peripheral mononuclear-myeloid-derived suppressor cells
PP2A	Protein phosphatase 2A
PPARs	Peroxisome-proliferator-activated receptors
PTEN	Phosphatase and tensin homolog
PTP1B	Protein tyrosine phosphatase 1B
PTPs	Protein tyrosine phosphatase
Ref-1	Redox factor-1
RES	Reactive electrophile species
RET	Reverse electron transfer
RNS	Reactive nitrogen species
ROCK	Rho-associated coiled-coil-containing protein kinase
ROS	Reactive oxygen species
RSS	Reactive sulfur species
SH2	Src homology 2
SHP2	Src homology 2 domain-containing protein tyrosine phosphatase 2
SMAD	Suppressor of mothers against decapentaplegic homolog
SOD	Superoxide dismutase
SSH-1L	Slingshot homolog-1 long
STAT	Signal transducer and activator of transcription
TCA	Tricarboxylic acid
TGF-β	Transforming growth factor-beta
Tiam	T-lymphoma invasion and metastasis-inducing protein
Trolox	6-Hydroxy-2,5,7,8-tetramethylchroman-2-carboxylic acid
Trx-1	Thioredoxin-1
TrxR	Thioredoxin reductase
WGA	Wheat germ agglutinin
ZEB1/2	Zinc finger E-box-binding homeobox 1/2

## References

[1] Dillekas H., Rogers M.S., Straume O. (2019). Are 90% of deaths from cancer caused by metastases?. Cancer Med..

[2] Leong L., Jandial R. (2013). Clinical Relevance of Targeting Cancer Metastases. Metastatic Cancer: Clinical and Biological Perspectives.

[3] Sies H. (2021). Oxidative eustress: On constant alert for redox homeostasis. Redox Biol..

[4] Sies H., Mailloux R.J., Jakob U. (2024). Author Correction: Fundamentals of redox regulation in biology. Nat. Rev. Mol. Cell Biol..

[5] Santolini J., Wootton S.A., Jackson A.A., Feelisch M. (2019). The Redox architecture of physiological function. Curr. Opin. Physiol..

[6] Holmstrom K.M., Finkel T. (2014). Cellular mechanisms and physiological consequences of redox-dependent signalling. Nat. Rev. Mol. Cell Biol..

[7] Tirichen H., Yaigoub H., Xu W., Wu C., Li R., Li Y. (2021). Mitochondrial Reactive Oxygen Species and Their Contribution in Chronic Kidney Disease Progression Through Oxidative Stress. Front. Physiol..

[8] de Almeida A., de Oliveira J., da Silva Pontes L.V., de Souza Junior J.F., Goncalves T.A.F., Dantas S.H., de Almeida Feitosa M.S., Silva A.O., de Medeiros I.A. (2022). ROS: Basic Concepts, Sources, Cellular Signaling, and its Implications in Aging Pathways. Oxidative Med. Cell. Longev..

[9] Snezhkina A.V., Kudryavtseva A.V., Kardymon O.L., Savvateeva M.V., Melnikova N.V., Krasnov G.S., Dmitriev A.A. (2019). ROS Generation and Antioxidant Defense Systems in Normal and Malignant Cells. Oxidative Med. Cell. Longev..

[10] Goetzman E., Gong Z., Zhang B., Muzumdar R. (2023). Complex II Biology in Aging, Health, and Disease. Antioxidants.

[11] Mailloux R.J. (2025). Targeted Redox Regulation α-Ketoglutarate Dehydrogenase Complex for the Treatment of Human Diseases. Cells.

[12] Magnani F., Mattevi A. (2019). Structure and mechanisms of ROS generation by NADPH oxidases. Curr. Opin. Struct. Biol..

[13] Fridovich I. (1995). Superoxide radical and superoxide dismutases. Annu. Rev. Biochem..

[14] Jomova K., Alomar S.Y., Alwasel S.H., Nepovimova E., Kuca K., Valko M. (2024). Several lines of antioxidant defense against oxidative stress: Antioxidant enzymes, nanomaterials with multiple enzyme-mimicking activities, and low-molecular-weight antioxidants. Arch. Toxicol..

[15] Shen H., Anastasio C. (2012). A Comparison of Hydroxyl Radical and Hydrogen Peroxide Generation in Ambient Particle Extracts and Laboratory Metal Solutions. Atmos. Environ..

[16] Hosios A.M., Vander Heiden M.G. (2018). The redox requirements of proliferating mammalian cells. J. Biol. Chem..

[17] Corkey B.E., Deeney J.T. (2020). The Redox Communication Network as a Regulator of Metabolism. Front. Physiol..

[18] Li B., Ming H., Qin S., Nice E.C., Dong J., Du Z., Huang C. (2025). Redox regulation: Mechanisms, biology and therapeutic targets in diseases. Signal Transduct. Target. Ther..

[19] Ray P.D., Huang B.W., Tsuji Y. (2012). Reactive oxygen species (ROS) homeostasis and redox regulation in cellular signaling. Cell. Signal..

[20] Franchina D.G., Dostert C., Brenner D. (2018). Reactive Oxygen Species: Involvement in T Cell Signaling and Metabolism. Trends Immunol..

[21] Wang K., Zhang T., Dong Q., Nice E.C., Huang C., Wei Y. (2013). Redox homeostasis: The linchpin in stem cell self-renewal and differentiation. Cell Death Dis..

[22] Tavassolifar M.J., Vodjgani M., Salehi Z., Izad M. (2020). The Influence of Reactive Oxygen Species in the Immune System and Pathogenesis of Multiple Sclerosis. Autoimmune Dis..

[23] Andreyev A.Y., Kushnareva Y.E., Starkova N.N., Starkov A.A. (2020). Metabolic ROS Signaling: To Immunity and Beyond. Biochemistry.

[24] Kaludercic N., Deshwal S., Di Lisa F. (2014). Reactive oxygen species and redox compartmentalization. Front. Physiol..

[25] Forrester S.J., Kikuchi D.S., Hernandes M.S., Xu Q., Griendling K.K. (2018). Reactive Oxygen Species in Metabolic and Inflammatory Signaling. Circ. Res..

[26] Jomova K., Raptova R., Alomar S.Y., Alwasel S.H., Nepovimova E., Kuca K., Valko M. (2023). Reactive oxygen species, toxicity, oxidative stress, and antioxidants: Chronic diseases and aging. Arch. Toxicol..

[27] Pacher P., Beckman J.S., Liaudet L. (2007). Nitric oxide and peroxynitrite in health and disease. Physiol. Rev..

[28] Cui S., Guo X., Wang S., Wei Z., Huang D., Zhang X., Zhu T.C., Huang Z. (2024). Singlet Oxygen in Photodynamic Therapy. Pharmaceuticals.

[29] Hovan A., Pevna V., Huntosova V., Miskovsky P., Bano G. (2024). Singlet oxygen lifetime changes in dying glioblastoma cells. Photochem. Photobiol..

[30] Fujii J., Soma Y., Matsuda Y. (2023). Biological Action of Singlet Molecular Oxygen from the Standpoint of Cell Signaling, Injury and Death. Molecules.

[31] Villalpando-Rodriguez G.E., Gibson S.B. (2021). Reactive Oxygen Species (ROS) Regulates Different Types of Cell Death by Acting as a Rheostat. Oxid. Med. Cell Longev..

[32] An X., Yu W., Liu J., Tang D., Yang L., Chen X. (2024). Oxidative cell death in cancer: Mechanisms and therapeutic opportunities. Cell Death Dis..

[33] Mittal M., Siddiqui M.R., Tran K., Reddy S.P., Malik A.B. (2014). Reactive oxygen species in inflammation and tissue injury. Antioxid. Redox Signal..

[34] Plociniczak A., Bukowska-Olech E., Wysocka E. (2025). The Complexity of Oxidative Stress in Human Age-Related Diseases-A Review. Metabolites.

[35] Le Lay S., Simard G., Martinez M.C., Andriantsitohaina R. (2014). Oxidative stress and metabolic pathologies: From an adipocentric point of view. Oxidative Med. Cell. Longev..

[36] Akhigbe R., Ajayi A. (2021). The impact of reactive oxygen species in the development of cardiometabolic disorders: A review. Lipids Health Dis..

[37] D’Oria R., Schipani R., Leonardini A., Natalicchio A., Perrini S., Cignarelli A., Laviola L., Giorgino F. (2020). The Role of Oxidative Stress in Cardiac Disease: From Physiological Response to Injury Factor. Oxidative Med. Cell. Longev..

[38] Dubois-Deruy E., Peugnet V., Turkieh A., Pinet F. (2020). Oxidative Stress in Cardiovascular Diseases. Antioxidants.

[39] Jomova K., Alomar S.Y., Valko R., Fresser L., Nepovimova E., Kuca K., Valko M. (2025). Interplay of oxidative stress and antioxidant mechanisms in cancer development and progression. Arch. Toxicol..

[40] Ursino C., Mouric C., Gros L., Bonnefoy N., Faget J. (2023). Intrinsic features of the cancer cell as drivers of immune checkpoint blockade response and refractoriness. Front. Immunol..

[41] Hanahan D. (2022). Hallmarks of Cancer: New Dimensions. Cancer Discov..

[42] Nieto M.A., Huang R.Y., Jackson R.A., Thiery J.P. (2016). Emt: 2016. Cell.

[43] Yang J., Antin P., Berx G., Blanpain C., Brabletz T., Bronner M., Campbell K., Cano A., Casanova J., Christofori G. (2021). Author Correction: Guidelines and definitions for research on epithelial-mesenchymal transition. Nat. Rev. Mol. Cell Biol..

[44] Roche J. (2018). The Epithelial-to-Mesenchymal Transition in Cancer. Cancers.

[45] Wei X., Liu R., Li W., Yu Q., Yang Q.T., Li T. (2025). Advances in research regarding epithelial-mesenchymal transition and prostate cancer. Front. Cell Dev. Biol..

[46] Gonzalez D.M., Medici D. (2014). Signaling mechanisms of the epithelial-mesenchymal transition. Sci. Signal..

[47] Celia-Terrassa T., Kang Y. (2024). How important is EMT for cancer metastasis?. PLoS Biol..

[48] Luond F., Sugiyama N., Bill R., Bornes L., Hager C., Tang F., Santacroce N., Beisel C., Ivanek R., Burglin T. (2021). Distinct contributions of partial and full EMT to breast cancer malignancy. Dev. Cell.

[49] Pastushenko I., Blanpain C. (2019). EMT Transition States during Tumor Progression and Metastasis. Trends Cell Biol..

[50] Pastushenko I., Brisebarre A., Sifrim A., Fioramonti M., Revenco T., Boumahdi S., Van Keymeulen A., Brown D., Moers V., Lemaire S. (2018). Identification of the tumour transition states occurring during EMT. Nature.

[51] Castaneda M., den Hollander P., Kuburich N.A., Rosen J.M., Mani S.A. (2022). Mechanisms of cancer metastasis. Semin. Cancer Biol..

[52] Parker A.L., Benguigui M., Fornetti J., Goddard E., Lucotti S., Insua-Rodriguez J., Wiegmans A.P., Early Career Leadership Council of the Metastasis Research Society (2022). Current challenges in metastasis research and future innovation for clinical translation. Clin. Exp. Metastasis.

[53] Lambert A.W., Pattabiraman D.R., Weinberg R.A. (2017). Emerging Biological Principles of Metastasis. Cell.

[54] Celia-Terrassa T., Kang Y. (2016). Distinctive properties of metastasis-initiating cells. Genes Dev..

[55] Sturgess V., Azubuike U.F., Tanner K. (2023). Vascular regulation of disseminated tumor cells during metastatic spread. Biophys. Rev..

[56] Kim K., Marquez-Palencia M., Malladi S. (2019). Metastatic Latency, a Veiled Threat. Front. Immunol..

[57] Eyles J., Puaux A.L., Wang X., Toh B., Prakash C., Hong M., Tan T.G., Zheng L., Ong L.C., Jin Y. (2010). Tumor cells disseminate early, but immunosurveillance limits metastatic outgrowth, in a mouse model of melanoma. J. Clin. Investig..

[58] Nishida N., Yano H., Nishida T., Kamura T., Kojiro M. (2006). Angiogenesis in cancer. Vasc. Health Risk Manag..

[59] Saman H., Raza S.S., Uddin S., Rasul K. (2020). Inducing Angiogenesis, a Key Step in Cancer Vascularization, and Treatment Approaches. Cancers.

[60] Azzi S., Hebda J.K., Gavard J. (2013). Vascular permeability and drug delivery in cancers. Front. Oncol..

[61] Lugano R., Ramachandran M., Dimberg A. (2020). Tumor angiogenesis: Causes, consequences, challenges and opportunities. Cell. Mol. Life Sci..

[62] Wechman S.L., Emdad L., Sarkar D., Das S.K., Fisher P.B. (2020). Vascular mimicry: Triggers, molecular interactions and in vivo models. Adv. Cancer Res..

[63] Kirschmann D.A., Seftor E.A., Hardy K.M., Seftor R.E., Hendrix M.J. (2012). Molecular pathways: Vasculogenic mimicry in tumor cells: Diagnostic and therapeutic implications. Clin. Cancer Res..

[64] Kim H.S., Won Y.J., Shim J.H., Kim H.J., Kim J., Hong H.N., Kim B.S. (2019). Morphological characteristics of vasculogenic mimicry and its correlation with EphA2 expression in gastric adenocarcinoma. Sci. Rep..

[65] Del Monte U. (2009). Does the cell number 10^9^ still really fit one gram of tumor tissue?. Cell Cycle.

[66] Scott J.G., Basanta D., Anderson A.R., Gerlee P. (2013). A mathematical model of tumour self-seeding reveals secondary metastatic deposits as drivers of primary tumour growth. J. R. Soc. Interface.

[67] Zhao Z.M., Zhao B., Bai Y., Iamarino A., Gaffney S.G., Schlessinger J., Lifton R.P., Rimm D.L., Townsend J.P. (2016). Early and multiple origins of metastatic lineages within primary tumors. Proc. Natl. Acad. Sci. USA.

[68] Friberg S., Nystrom A. (2015). Cancer Metastases: Early Dissemination and Late Recurrences. Cancer Growth Metastasis.

[69] Hu Z., Curtis C. (2020). Looking backward in time to define the chronology of metastasis. Nat. Commun..

[70] Williams A.L., Fitzgerald J.E., Ivich F., Sontag E.D., Niedre M. (2020). Short-Term Circulating Tumor Cell Dynamics in Mouse Xenograft Models and Implications for Liquid Biopsy. Front. Oncol..

[71] Kurma K., Alix-Panabieres C. (2023). Mechanobiology and survival strategies of circulating tumor cells: A process towards the invasive and metastatic phenotype. Front. Cell Dev. Biol..

[72] Fidler I.J. (1970). Metastasis: Quantitative analysis of distribution and fate of tumor emboli labeled with 125 I-5-iodo-2′-deoxyuridine. J. Natl. Cancer Inst..

[73] Luzzi K.J., MacDonald I.C., Schmidt E.E., Kerkvliet N., Morris V.L., Chambers A.F., Groom A.C. (1998). Multistep nature of metastatic inefficiency: Dormancy of solitary cells after successful extravasation and limited survival of early micrometastases. Am. J. Pathol..

[74] Amintas S., Bedel A., Moreau-Gaudry F., Boutin J., Buscail L., Merlio J.P., Vendrely V., Dabernat S., Buscail E. (2020). Circulating Tumor Cell Clusters: United We Stand Divided We Fall. Int. J. Mol. Sci..

[75] Aceto N., Bardia A., Miyamoto D.T., Donaldson M.C., Wittner B.S., Spencer J.A., Yu M., Pely A., Engstrom A., Zhu H. (2014). Circulating tumor cell clusters are oligoclonal precursors of breast cancer metastasis. Cell.

[76] Cho E.H., Wendel M., Luttgen M., Yoshioka C., Marrinucci D., Lazar D., Schram E., Nieva J., Bazhenova L., Morgan A. (2012). Characterization of circulating tumor cell aggregates identified in patients with epithelial tumors. Phys. Biol..

[77] Cheung K.J., Padmanaban V., Silvestri V., Schipper K., Cohen J.D., Fairchild A.N., Gorin M.A., Verdone J.E., Pienta K.J., Bader J.S. (2016). Polyclonal breast cancer metastases arise from collective dissemination of keratin 14-expressing tumor cell clusters. Proc. Natl. Acad. Sci. USA.

[78] Schuster E., Taftaf R., Reduzzi C., Albert M.K., Romero-Calvo I., Liu H. (2021). Better together: Circulating tumor cell clustering in metastatic cancer. Trends Cancer.

[79] Ju J.A., Lee C.J., Thompson K.N., Ory E.C., Lee R.M., Mathias T.J., Pratt S.J.P., Vitolo M.I., Jewell C.M., Martin S.S. (2020). Partial thermal imidization of polyelectrolyte multilayer cell tethering surfaces (TetherChip) enables efficient cell capture and microtentacle fixation for circulating tumor cell analysis. Lab Chip.

[80] Reader J.C., Fan C., Ory E.C., Ju J., Lee R., Vitolo M.I., Smith P., Wu S., Ching M.M.N., Asiedu E.B. (2022). Microtentacle Formation in Ovarian Carcinoma. Cancers.

[81] Balzer E.M., Whipple R.A., Thompson K., Boggs A.E., Slovic J., Cho E.H., Matrone M.A., Yoneda T., Mueller S.C., Martin S.S. (2010). c-Src differentially regulates the functions of microtentacles and invadopodia. Oncogene.

[82] Bhandary L., Whipple R.A., Vitolo M.I., Charpentier M.S., Boggs A.E., Chakrabarti K.R., Thompson K.N., Martin S.S. (2015). ROCK inhibition promotes microtentacles that enhance reattachment of breast cancer cells. Oncotarget.

[83] Boggs A.E., Vitolo M.I., Whipple R.A., Charpentier M.S., Goloubeva O.G., Ioffe O.B., Tuttle K.C., Slovic J., Lu Y., Mills G.B. (2015). α-Tubulin acetylation elevated in metastatic and basal-like breast cancer cells promotes microtentacle formation, adhesion, and invasive migration. Cancer Res..

[84] Charpentier M.S., Whipple R.A., Vitolo M.I., Boggs A.E., Slovic J., Thompson K.N., Bhandary L., Martin S.S. (2014). Curcumin targets breast cancer stem-like cells with microtentacles that persist in mammospheres and promote reattachment. Cancer Res..

[85] Matrone M.A., Whipple R.A., Balzer E.M., Martin S.S. (2010). Microtentacles tip the balance of cytoskeletal forces in circulating tumor cells. Cancer Res..

[86] Whipple R.A., Balzer E.M., Cho E.H., Matrone M.A., Yoon J.R., Martin S.S. (2008). Vimentin filaments support extension of tubulin-based microtentacles in detached breast tumor cells. Cancer Res..

[87] Whipple R.A., Cheung A.M., Martin S.S. (2007). Detyrosinated microtubule protrusions in suspended mammary epithelial cells promote reattachment. Exp. Cell Res..

[88] Vitolo M.I., Boggs A.E., Whipple R.A., Yoon J.R., Thompson K., Matrone M.A., Cho E.H., Balzer E.M., Martin S.S. (2013). Loss of PTEN induces microtentacles through PI3K-independent activation of cofilin. Oncogene.

[89] Yoon J.R., Whipple R.A., Balzer E.M., Cho E.H., Matrone M.A., Peckham M., Martin S.S. (2010). Local anesthetics inhibit kinesin motility and microtentacle protrusions in human epithelial and breast tumor cells. Breast Cancer Res. Treat..

[90] Thompson K.N., Ju J.A., Ory E.C., Pratt S.J.P., Lee R.M., Mathias T.J., Chang K.T., Lee C.J., Goloubeva O.G., Bailey P.C. (2022). Microtubule disruption reduces metastasis more effectively than primary tumor growth. Breast Cancer Res..

[91] Schiliro C., Firestein B.L. (2021). Mechanisms of Metabolic Reprogramming in Cancer Cells Supporting Enhanced Growth and Proliferation. Cells.

[92] Zhao Y., Ye X., Xiong Z., Ihsan A., Ares I., Martinez M., Lopez-Torres B., Martinez-Larranaga M.R., Anadon A., Wang X. (2023). Cancer Metabolism: The Role of ROS in DNA Damage and Induction of Apoptosis in Cancer Cells. Metabolites.

[93] Perillo B., Di Donato M., Pezone A., Di Zazzo E., Giovannelli P., Galasso G., Castoria G., Migliaccio A. (2020). ROS in cancer therapy: The bright side of the moon. Exp. Mol. Med..

[94] Chandel N.S., Maltepe E., Goldwasser E., Mathieu C.E., Simon M.C., Schumacker P.T. (1998). Mitochondrial reactive oxygen species trigger hypoxia-induced transcription. Proc. Natl. Acad. Sci. USA.

[95] Oh A., Pardo M., Rodriguez A., Yu C., Nguyen L., Liang O., Chorzalska A., Dubielecka P.M. (2023). NF-κB signaling in neoplastic transition from epithelial to mesenchymal phenotype. Cell Commun. Signal..

[96] Jiang J., Wang K., Chen Y., Chen H., Nice E.C., Huang C. (2017). Redox regulation in tumor cell epithelial-mesenchymal transition: Molecular basis and therapeutic strategy. Signal Transduct. Target. Ther..

[97] Cichon M.A., Radisky D.C. (2014). ROS-induced epithelial-mesenchymal transition in mammary epithelial cells is mediated by NF-kB-dependent activation of Snail. Oncotarget.

[98] Radisky D.C., Levy D.D., Littlepage L.E., Liu H., Nelson C.M., Fata J.E., Leake D., Godden E.L., Albertson D.G., Nieto M.A. (2005). Rac1b and reactive oxygen species mediate MMP-3-induced EMT and genomic instability. Nature.

[99] Ma M., Shi F., Zhai R., Wang H., Li K., Xu C., Yao W., Zhou F. (2021). TGF-β promote epithelial-mesenchymal transition via NF-κB/NOX4/ROS signal pathway in lung cancer cells. Mol. Biol. Rep..

[100] Su X., Yang Y., Guo C., Zhang R., Sun S., Wang Y., Qiao Q., Fu Y., Pang Q. (2021). NOX4-Derived ROS Mediates TGF-*β*1-Induced Metabolic Reprogramming during Epithelial-Mesenchymal Transition through the PI3K/AKT/HIF-1*α* Pathway in Glioblastoma. Oxidative Med. Cell. Longev..

[101] Nisimoto Y., Diebold B.A., Cosentino-Gomes D., Lambeth J.D. (2014). Nox4: A hydrogen peroxide-generating oxygen sensor. Biochemistry.

[102] Boudreau H.E., Casterline B.W., Rada B., Korzeniowska A., Leto T.L. (2012). Nox4 involvement in TGF-beta and SMAD3-driven induction of the epithelial-to-mesenchymal transition and migration of breast epithelial cells. Free Radic. Biol. Med..

[103] Qutub A.A., Popel A.S. (2007). Three autocrine feedback loops determine HIF1α expression in chronic hypoxia. Biochim. Biophys. Acta.

[104] Yang J., Zhang X., Zhang Y., Zhu D., Zhang L., Li Y., Zhu Y., Li D., Zhou J. (2016). HIF-2α promotes epithelial-mesenchymal transition through regulating Twist2 binding to the promoter of E-cadherin in pancreatic cancer. J. Exp. Clin. Cancer Res..

[105] Zhang K.D., Hu B., Cen G., Yang Y.H., Chen W.W., Guo Z.Y., Wang X.F., Zhao Q., Qiu Z.J. (2020). MiR-301a transcriptionally activated by HIF-2α promotes hypoxia-induced epithelial-mesenchymal transition by targeting TP63 in pancreatic cancer. World J. Gastroenterol..

[106] Villareal L.B., Falcon D.M., Xie L., Xue X. (2024). Hypoxia-inducible factor 3α1 increases epithelial-to-mesenchymal transition and iron uptake to drive colorectal cancer liver metastasis. Br. J. Cancer.

[107] Lodish H., Berk A., Matsudaira P., Kaiser C.A., Krieger M., Scott M.P., Zipursky S.L., Darnell J. (2010). Molecular Cell Biology.

[108] Yang M.H., Wu M.Z., Chiou S.H., Chen P.M., Chang S.Y., Liu C.J., Teng S.C., Wu K.J. (2008). Direct regulation of TWIST by HIF-1α promotes metastasis. Nat. Cell Biol..

[109] Zhang L., Huang G., Li X., Zhang Y., Jiang Y., Shen J., Liu J., Wang Q., Zhu J., Feng X. (2013). Hypoxia induces epithelial-mesenchymal transition via activation of SNAI1 by hypoxia-inducible factor -1α in hepatocellular carcinoma. BMC Cancer.

[110] Luo D., Wang J., Li J., Post M. (2011). Mouse snail is a target gene for HIF. Mol. Cancer Res..

[111] Zhang W., Shi X., Peng Y., Wu M., Zhang P., Xie R., Wu Y., Yan Q., Liu S., Wang J. (2015). HIF-1α Promotes Epithelial-Mesenchymal Transition and Metastasis through Direct Regulation of ZEB1 in Colorectal Cancer. PLoS ONE.

[112] Liu J., Huang B., Xiu Z., Zhou Z., Liu J., Li X., Tang X. (2018). PI3K/Akt/HIF-1α signaling pathway mediates HPV-16 oncoprotein-induced expression of EMT-related transcription factors in non-small cell lung cancer cells. J. Cancer.

[113] Schieber M., Chandel N.S. (2014). ROS function in redox signaling and oxidative stress. Curr. Biol..

[114] Lee S.R., Yang K.S., Kwon J., Lee C., Jeong W., Rhee S.G. (2002). Reversible inactivation of the tumor suppressor PTEN by H_2_O_2_. J. Biol. Chem..

[115] Lee S.R., Kwon K.S., Kim S.R., Rhee S.G. (1998). Reversible inactivation of protein-tyrosine phosphatase 1B in A431 cells stimulated with epidermal growth factor. J. Biol. Chem..

[116] Meng T.C., Fukada T., Tonks N.K. (2002). Reversible oxidation and inactivation of protein tyrosine phosphatases in vivo. Mol. Cell.

[117] Chiarugi P. (2003). Reactive oxygen species as mediators of cell adhesion. Ital. J. Biochem..

[118] Frijhoff J., Dagnell M., Godfrey R., Ostman A. (2014). Regulation of protein tyrosine phosphatase oxidation in cell adhesion and migration. Antioxid. Redox Signal..

[119] Welsh C.L., Madan L.K. (2024). Protein Tyrosine Phosphatase regulation by Reactive Oxygen Species. Adv. Cancer Res..

[120] Chen J., Li W., Zhang C., Wen D., Jiao C. (2024). Tyrosine phosphatase SHP2 promoted the progression of CRC via modulating the PI3K/BRD4/TFEB signaling induced ferroptosis. Discov. Oncol..

[121] Acevedo A., Gonzalez-Billault C. (2018). Crosstalk between Rac1-mediated actin regulation and ROS production. Free Radic. Biol. Med..

[122] Liang J., Oyang L., Rao S., Han Y., Luo X., Yi P., Lin J., Xia L., Hu J., Tan S. (2021). Rac1, A Potential Target for Tumor Therapy. Front. Oncol..

[123] Hobbs G.A., Zhou B., Cox A.D., Campbell S.L. (2014). Rho GTPases, oxidation, and cell redox control. Small GTPases.

[124] Baba R.A., Mir H.A., Mokhdomi T.A., Bhat H.F., Ahmad A., Khanday F.A. (2024). Quercetin suppresses ROS production and migration by specifically targeting Rac1 activation in gliomas. Front. Pharmacol..

[125] Xu Q., Huff L.P., Fujii M., Griendling K.K. (2017). Redox regulation of the actin cytoskeleton and its role in the vascular system. Free Radic. Biol. Med..

[126] Kim J.S., Huang T.Y., Bokoch G.M. (2009). Reactive oxygen species regulate a slingshot-cofilin activation pathway. Mol. Biol. Cell.

[127] Samstag Y., John I., Wabnitz G.H. (2013). Cofilin: A redox sensitive mediator of actin dynamics during T-cell activation and migration. Immunol. Rev..

[128] Cameron J.M., Gabrielsen M., Chim Y.H., Munro J., McGhee E.J., Sumpton D., Eaton P., Anderson K.I., Yin H., Olson M.F. (2015). Polarized cell motility induces hydrogen peroxide to inhibit cofilin via cysteine oxidation. Curr. Biol..

[129] Balta E., Hardt R., Liang J., Kirchgessner H., Orlik C., Jahraus B., Hillmer S., Meuer S., Hubner K., Wabnitz G.H. (2019). Spatial oxidation of L-plastin downmodulates actin-based functions of tumor cells. Nat. Commun..

[130] Valdivia A., Duran C., Lee M., Williams H.C., Lee M.Y., San Martin A. (2023). Nox1-based NADPH oxidase regulates the Par protein complex activity to control cell polarization. Front. Cell Dev. Biol..

[131] Richter S.M., Massman L.C., Stuehr D.J., Sweeny E.A. (2023). Functional interactions between NADPH oxidase 5 and actin. Front. Cell Dev. Biol..

[132] Taddei M.L., Chiarugi P., Cirri P., Buricchi F., Fiaschi T., Giannoni E., Talini D., Cozzi G., Formigli L., Raugei G. (2002). β-catenin interacts with low-molecular-weight protein tyrosine phosphatase leading to cadherin-mediated cell-cell adhesion increase. Cancer Res..

[133] Rao R.K., Basuroy S., Rao V.U., Karnaky K.J., Gupta A. (2002). Tyrosine phosphorylation and dissociation of occludin-ZO-1 and E-cadherin-β-catenin complexes from the cytoskeleton by oxidative stress. Biochem. J..

[134] Chen Y.H., Hsu J.Y., Chu C.T., Chang Y.W., Fan J.R., Yang M.H., Chen H.C. (2023). Loss of cell-cell adhesion triggers cell migration through Rac1-dependent ROS generation. Life Sci. Alliance.

[135] Matrullo G., Filomeni G., Rizza S. (2025). Redox regulation of focal adhesions. Redox Biol..

[136] Kim D.H., Wirtz D. (2013). Focal adhesion size uniquely predicts cell migration. FASEB J..

[137] Heppner D.E. (2021). Structural insights into redox-active cysteine residues of the Src family kinases. Redox Biol..

[138] Westhoff M.A., Serrels B., Fincham V.J., Frame M.C., Carragher N.O. (2004). SRC-mediated phosphorylation of focal adhesion kinase couples actin and adhesion dynamics to survival signaling. Mol. Cell. Biol..

[139] Nagano M., Hoshino D., Koshikawa N., Akizawa T., Seiki M. (2012). Turnover of focal adhesions and cancer cell migration. Int. J. Cell Biol..

[140] Zhao J., Guan J.L. (2009). Signal transduction by focal adhesion kinase in cancer. Cancer Metastasis Rev..

[141] Irby R.B., Yeatman T.J. (2000). Role of Src expression and activation in human cancer. Oncogene.

[142] Shinohara M., Adachi Y., Mitsushita J., Kuwabara M., Nagasawa A., Harada S., Furuta S., Zhang Y., Seheli K., Miyazaki H. (2010). Reactive oxygen generated by NADPH oxidase 1 (Nox1) contributes to cell invasion by regulating matrix metalloprotease-9 production and cell migration. J. Biol. Chem..

[143] Shi Y., An D., Liu Y., Feng Q., Fang X., Pan G., Wang Q. (2017). Propoxur enhances MMP-2 expression and the corresponding invasion of human breast cancer cells via the ERK/Nrf2 signaling pathway. Oncotarget.

[144] Lee G.H., Jin S.W., Kim S.J., Pham T.H., Choi J.H., Jeong H.G. (2019). Tetrabromobisphenol A Induces MMP-9 Expression via NADPH Oxidase and the activation of ROS, MAPK, and Akt Pathways in Human Breast Cancer MCF-7 Cells. Toxicol. Res..

[145] Sun M., Hong S., Li W., Wang P., You J., Zhang X., Tang F., Wang P., Zhang C. (2016). MiR-99a regulates ROS-mediated invasion and migration of lung adenocarcinoma cells by targeting NOX4. Oncol. Rep..

[146] Yamazaki S., Miyoshi N., Kawabata K., Yasuda M., Shimoi K. (2014). Quercetin-3-*O*-glucuronide inhibits noradrenaline-promoted invasion of MDA-MB-231 human breast cancer cells by blocking β_2_-adrenergic signaling. Arch. Biochem. Biophys..

[147] Wang P., Zeng Y., Liu T., Zhang C., Yu P.W., Hao Y.X., Luo H.X., Liu G. (2014). Chloride intracellular channel 1 regulates colon cancer cell migration and invasion through ROS/ERK pathway. World J. Gastroenterol..

[148] Gallelli L., Falcone D., Scaramuzzino M., Pelaia G., D’Agostino B., Mesuraca M., Terracciano R., Spaziano G., Maselli R., Navarra M. (2014). Effects of simvastatin on cell viability and proinflammatory pathways in lung adenocarcinoma cells exposed to hydrogen peroxide. BMC Pharmacol. Toxicol..

[149] Riemann A., Schneider B., Ihling A., Nowak M., Sauvant C., Thews O., Gekle M. (2011). Acidic environment leads to ROS-induced MAPK signaling in cancer cells. PLoS ONE.

[150] Weinberg F., Chandel N.S. (2009). Reactive oxygen species-dependent signaling regulates cancer. Cell. Mol. Life Sci..

[151] Brandl N., Seitz R., Sendtner N., Muller M., Gulow K. (2025). Living on the Edge: ROS Homeostasis in Cancer Cells and Its Potential as a Therapeutic Target. Antioxidants.

[152] Xia Y., Lian S., Khoi P.N., Yoon H.J., Joo Y.E., Chay K.O., Kim K.K., Do Jung Y. (2015). Chrysin inhibits tumor promoter-induced MMP-9 expression by blocking AP-1 via suppression of ERK and JNK pathways in gastric cancer cells. PLoS ONE.

[153] Morgan M.J., Liu Z.G. (2011). Crosstalk of reactive oxygen species and NF-κB signaling. Cell Res..

[154] Malcomson E., Zhang W. (2024). ROS-Mediated Upregulation of MMP9 Expression via MAPK-AP1 Signaling Pathway and Disruption of Blood-Brain Barrier. Austin J. Cerebrovasc. Dis. Stroke.

[155] Hsieh H.L., Wang H.H., Wu W.B., Chu P.J., Yang C.M. (2010). Transforming growth factor-β1 induces matrix metalloproteinase-9 and cell migration in astrocytes: Roles of ROS-dependent ERK- and JNK-NF-κB pathways. J. Neuroinflamm..

[156] Vincenti M.P., Brinckerhoff C.E. (2007). Signal transduction and cell-type specific regulation of matrix metalloproteinase gene expression: Can MMPs be good for you?. J. Cell. Physiol..

[157] Bian Y., Xiang Z., Wang Y., Ren Q., Chen G., Xiang B., Wang J., Zhang C., Pei S., Guo S. (2023). Immunomodulatory roles of metalloproteinases in rheumatoid arthritis. Front. Pharmacol..

[158] Bergman M.R., Cheng S., Honbo N., Piacentini L., Karliner J.S., Lovett D.H. (2003). A functional activating protein 1 (AP-1) site regulates matrix metalloproteinase 2 (MMP-2) transcription by cardiac cells through interactions with JunB-Fra1 and JunB-FosB heterodimers. Biochem. J..

[159] Song H., Ki S.H., Kim S.G., Moon A. (2006). Activating transcription factor 2 mediates matrix metalloproteinase-2 transcriptional activation induced by p38 in breast epithelial cells. Cancer Res..

[160] Wan R., Mo Y., Chien S., Li Y., Li Y., Tollerud D.J., Zhang Q. (2011). The role of hypoxia inducible factor-1α in the increased MMP-2 and MMP-9 production by human monocytes exposed to nickel nanoparticles. Nanotoxicology.

[161] Bhoumik A., Lopez-Bergami P., Ronai Z. (2007). ATF2 on the double—Activating transcription factor and DNA damage response protein. Pigment Cell Res..

[162] Wu Y., Zhang J., Li C., Hu H., Qin B., Wang T., Lu Y., Wang S. (2021). The Activation of ROS/NF-*κ*B/MMP-9 Pathway Promotes Calcium-Induced Kidney Crystal Deposition. Oxidative Med. Cell. Longev..

[163] Yang J., Wei D., Liu J. (2005). Repressions of MMP-9 expression and NF-κB localization are involved in inhibition of lung carcinoma 95-D cell invasion by (–)-epigallocatechin-3-gallate. Biomed. Pharmacother..

[164] Choi J.Y., Jang Y.S., Min S.Y., Song J.Y. (2011). Overexpression of MMP-9 and HIF-1α in Breast Cancer Cells under Hypoxic Conditions. J. Breast Cancer.

[165] Ortiz-Barahona A., Villar D., Pescador N., Amigo J., del Peso L. (2010). Genome-wide identification of hypoxia-inducible factor binding sites and target genes by a probabilistic model integrating transcription-profiling data and in silico binding site prediction. Nucleic Acids Res..

[166] Mahara S., Lee P.L., Feng M., Tergaonkar V., Chng W.J., Yu Q. (2016). HIFI-α activation underlies a functional switch in the paradoxical role of Ezh2/PRC2 in breast cancer. Proc. Natl. Acad. Sci. USA.

[167] Zare Z., Dizaj T.N., Lohrasbi A., Sheikhalishahi Z.S., Asadi A., Zakeri M., Hosseinabadi F., Abazari O., Abbasi M., Khanicheragh P. (2021). Silibinin Inhibits TGF-β-induced MMP-2 and MMP-9 Through Smad Signaling Pathway in Colorectal Cancer HT-29 Cells. Basic. Clin. Cancer Res..

[168] Li S., Ung T.T., Nguyen T.T., Sah D.K., Park S.Y., Jung Y.D. (2020). Cholic Acid Stimulates MMP-9 in Human Colon Cancer Cells via Activation of MAPK, AP-1, and NF-κB Activity. Int. J. Mol. Sci..

[169] Mori K., Uchida T., Yoshie T., Mizote Y., Ishikawa F., Katsuyama M., Shibanuma M. (2019). A mitochondrial ROS pathway controls matrix metalloproteinase 9 levels and invasive properties in RAS-activated cancer cells. FEBS J..

[170] Van Wart H.E., Birkedal-Hansen H. (1990). The cysteine switch: A principle of regulation of metalloproteinase activity with potential applicability to the entire matrix metalloproteinase gene family. Proc. Natl. Acad. Sci. USA.

[171] Dujon A.M., Capp J.P., Brown J.S., Pujol P., Gatenby R.A., Ujvari B., Alix-Panabieres C., Thomas F. (2021). Is There One Key Step in the Metastatic Cascade?. Cancers.

[172] Adeshakin F.O., Adeshakin A.O., Afolabi L.O., Yan D., Zhang G., Wan X. (2021). Mechanisms for Modulating Anoikis Resistance in Cancer and the Relevance of Metabolic Reprogramming. Front. Oncol..

[173] Graham K.A., Kulawiec M., Owens K.M., Li X., Desouki M.M., Chandra D., Singh K.K. (2010). NADPH oxidase 4 is an oncoprotein localized to mitochondria. Cancer Biol. Ther..

[174] Kim H., Sung J.Y., Park E.K., Kho S., Koo K.H., Park S.Y., Goh S.H., Jeon Y.K., Oh S., Park B.K. (2017). Regulation of anoikis resistance by NADPH oxidase 4 and epidermal growth factor receptor. Br. J. Cancer.

[175] Du S., Miao J., Zhu Z., Xu E., Shi L., Ai S., Wang F., Kang X., Chen H., Lu X. (2018). NADPH oxidase 4 regulates anoikis resistance of gastric cancer cells through the generation of reactive oxygen species and the induction of EGFR. Cell Death Dis..

[176] Lin X.L., Yang L., Fu S.W., Lin W.F., Gao Y.J., Chen H.Y., Ge Z.Z. (2017). Overexpression of NOX4 predicts poor prognosis and promotes tumor progression in human colorectal cancer. Oncotarget.

[177] Vaquero E.C., Edderkaoui M., Pandol S.J., Gukovsky I., Gukovskaya A.S. (2004). Reactive oxygen species produced by NAD(P)H oxidase inhibit apoptosis in pancreatic cancer cells. J. Biol. Chem..

[178] Chen Y.H., Chien C.Y., Fang F.M., Huang T.L., Su Y.Y., Luo S.D., Huang C.C., Lin W.C., Li S.H. (2018). Nox4 Overexpression as a Poor Prognostic Factor in Patients with Oral Tongue Squamous Cell Carcinoma Receiving Surgical Resection. J. Clin. Med..

[179] Mir S., Ormsbee Golden B.D., Griess B.J., Vengoji R., Tom E., Kosmacek E.A., Oberley-Deegan R.E., Talmon G.A., Band V., Teoh-Fitzgerald M.L. (2022). Upregulation of Nox4 induces a pro-survival Nrf2 response in cancer-associated fibroblasts that promotes tumorigenesis and metastasis, in part via Birc5 induction. Breast Cancer Res..

[180] Szczerba B.M., Castro-Giner F., Vetter M., Krol I., Gkountela S., Landin J., Scheidmann M.C., Donato C., Scherrer R., Singer J. (2019). Neutrophils escort circulating tumour cells to enable cell cycle progression. Nature.

[181] Ju J.A., Thompson K.N., Annis D.A., Mull M.L., Gilchrist D.E., Moriarty A., Chang K.T., Stemberger M.B., Noto M.J., Vitolo M.I. (2025). Tubulin-Based Microtentacles Aid in Heterotypic Clustering of Neutrophil-Differentiated HL-60 Cells and Breast Tumor Cells. Adv. Sci..

[182] Sprouse M.L., Welte T., Boral D., Liu H.N., Yin W., Vishnoi M., Goswami-Sewell D., Li L., Pei G., Jia P. (2019). PMN-MDSCs Enhance CTC Metastatic Properties through Reciprocal Interactions via ROS/Notch/Nodal Signaling. Int. J. Mol. Sci..

[183] Han H.J., Sung J.Y., Kim S.H., Yun U.J., Kim H., Jang E.J., Yoo H.E., Hong E.K., Goh S.H., Moon A. (2021). Fibronectin regulates anoikis resistance via cell aggregate formation. Cancer Lett..

[184] Wang Y., Liu L., Zhang X., Liang T., Bai X. (2025). Cancer dormancy and metabolism: From molecular insights to translational opportunities. Cancer Lett..

[185] Vomund S., Schafer A., Parnham M.J., Brune B., von Knethen A. (2017). Nrf2, the Master Regulator of Anti-Oxidative Responses. Int. J. Mol. Sci..

[186] Ghoneum A., Abdulfattah A.Y., Warren B.O., Shu J., Said N. (2020). Redox Homeostasis and Metabolism in Cancer: A Complex Mechanism and Potential Targeted Therapeutics. Int. J. Mol. Sci..

[187] Fox D.B., Garcia N.M.G., McKinney B.J., Lupo R., Noteware L.C., Newcomb R., Liu J., Locasale J.W., Hirschey M.D., Alvarez J.V. (2020). NRF2 activation promotes the recurrence of dormant tumour cells through regulation of redox and nucleotide metabolism. Nat. Metab..

[188] Eggler A.L., Small E., Hannink M., Mesecar A.D. (2009). Cul3-mediated Nrf2 ubiquitination and antioxidant response element (ARE) activation are dependent on the partial molar volume at position 151 of Keap1. Biochem. J..

[189] Jiang L., Shestov A.A., Swain P., Yang C., Parker S.J., Wang Q.A., Terada L.S., Adams N.D., McCabe M.T., Pietrak B. (2016). Reductive carboxylation supports redox homeostasis during anchorage-independent growth. Nature.

[190] Risson E., Nobre A.R., Maguer-Satta V., Aguirre-Ghiso J.A. (2020). The current paradigm and challenges ahead for the dormancy of disseminated tumor cells. Nat. Cancer.

[191] Schafer Z.T., Grassian A.R., Song L., Jiang Z., Gerhart-Hines Z., Irie H.Y., Gao S., Puigserver P., Brugge J.S. (2009). Antioxidant and oncogene rescue of metabolic defects caused by loss of matrix attachment. Nature.

[192] Davison C.A., Durbin S.M., Thau M.R., Zellmer V.R., Chapman S.E., Diener J., Wathen C., Leevy W.M., Schafer Z.T. (2013). Antioxidant enzymes mediate survival of breast cancer cells deprived of extracellular matrix. Cancer Res..

[193] Teng T., Kamal M., Iriondo O., Amzaleg Y., Luo C., Thomas A., Lee G., Hsu C.J., Nguyen J.D., Kang I. (2021). N-Acetyl-L-cysteine Promotes Ex Vivo Growth and Expansion of Single Circulating Tumor Cells by Mitigating Cellular Stress Responses. Mol. Cancer Res..

[194] Jalmukhambetova A., Baltabekova A., Tolebay A., Rakhimgerey N., Molnar F., Thanh Pham T., Burska A.N., Sarbassov D.D. (2025). Oxidative stress induces cortical stiffening and cytoskeletal remodelling in pre-apoptotic cancer cells. Cell Stress.

[195] Bamburg J.R., Minamide L.S., Wiggan O., Tahtamouni L.H., Kuhn T.B. (2021). Cofilin and Actin Dynamics: Multiple Modes of Regulation and Their Impacts in Neuronal Development and Degeneration. Cells.

[196] Huang R., Chen H., Liang J., Li Y., Yang J., Luo C., Tang Y., Ding Y., Liu X., Yuan Q. (2021). Dual Role of Reactive Oxygen Species and their Application in Cancer Therapy. J. Cancer.

[197] Dikalov S.I., Harrison D.G. (2014). Methods for detection of mitochondrial and cellular reactive oxygen species. Antioxid. Redox Signal..

[198] Hom L.M., Schafer Z.T. (2023). Assessment of Metabolic Pathways and Parameters in Extracellular Matrix-Detached Cells. Methods Mol. Biol..

[199] Balzer E.M., Whipple R.A., Cho E.H., Matrone M.A., Martin S.S. (2010). Antimitotic chemotherapeutics promote adhesive responses in detached and circulating tumor cells. Breast Cancer Res. Treat..

[200] Chang K.T., Thompson K.N., Pratt S.J.P., Ju J.A., Lee R.M., Mathias T.J., Mull M.L., Annis D.A., Ory E.C., Stemberger M.B. (2023). Elevation of Cytoplasmic Calcium Suppresses Microtentacle Formation and Function in Breast Tumor Cells. Cancers.

[201] Matrone M.A., Whipple R.A., Thompson K., Cho E.H., Vitolo M.I., Balzer E.M., Yoon J.R., Ioffe O.B., Tuttle K.C., Tan M. (2010). Metastatic breast tumors express increased tau, which promotes microtentacle formation and the reattachment of detached breast tumor cells. Oncogene.

[202] Stemberger M.B., Ju J.A., Thompson K.N., Mathias T.J., Jerrett A.E., Chang K.T., Ory E.C., Annis D.A., Mull M.L., Gilchrist D.E. (2023). Hydrogen Peroxide Induces alpha-Tubulin Detyrosination and Acetylation and Impacts Breast Cancer Metastatic Phenotypes. Cells.

[203] Whipple R.A., Matrone M.A., Cho E.H., Balzer E.M., Vitolo M.I., Yoon J.R., Ioffe O.B., Tuttle K.C., Yang J., Martin S.S. (2010). Epithelial-to-mesenchymal transition promotes tubulin detyrosination and microtentacles that enhance endothelial engagement. Cancer Res..

[204] Whipple R.A., Vitolo M.I., Boggs A.E., Charpentier M.S., Thompson K., Martin S.S. (2013). Parthenolide and costunolide reduce microtentacles and tumor cell attachment by selectively targeting detyrosinated tubulin independent from NF-κB inhibition. Breast Cancer Res..

[205] Weber J., Bollepalli L., Belenguer A.M., Antonio M.D., De Mitri N., Joseph J., Balasubramanian S., Hunter C.A., Bohndiek S.E. (2019). An Activatable Cancer-Targeted Hydrogen Peroxide Probe for Photoacoustic and Fluorescence Imaging. Cancer Res..

[206] Van de Bittner G.C., Dubikovskaya E.A., Bertozzi C.R., Chang C.J. (2010). In vivo imaging of hydrogen peroxide production in a murine tumor model with a chemoselective bioluminescent reporter. Proc. Natl. Acad. Sci. USA.

[207] Boutagy N.E., Wu J., Cai Z., Zhang W., Booth C.J., Kyriakides T.C., Pfau D., Mulnix T., Liu Z., Miller E.J. (2018). In Vivo Reactive Oxygen Species Detection with a Novel Positron Emission Tomography Tracer, ^18^F-DHMT, Allows for Early Detection of Anthracycline-Induced Cardiotoxicity in Rodents. JACC Basic Transl. Sci..

[208] Dos Reis Oliveira C., Pereira J.C., Barros Ibiapina A., Roseno Martins I.R., de Castro E.S.J.M., Ferreira P.M.P., Carneiro da Silva F.C. (2023). Buthionine sulfoximine and chemoresistance in cancer treatments: A systematic review with meta-analysis of preclinical studies. J. Toxicol. Environ. Health B Crit. Rev..

[209] Kannappan V., Ali M., Small B., Rajendran G., Elzhenni S., Taj H., Wang W., Dou Q.P. (2021). Recent Advances in Repurposing Disulfiram and Disulfiram Derivatives as Copper-Dependent Anticancer Agents. Front. Mol. Biosci..

[210] Lin J., Zahurak M., Beer T.M., Ryan C.J., Wilding G., Mathew P., Morris M., Callahan J.A., Gordon G., Reich S.D. (2013). A non-comparative randomized phase II study of 2 doses of ATN-224, a copper/zinc superoxide dismutase inhibitor, in patients with biochemically recurrent hormone-naive prostate cancer. Urol. Oncol..

[211] O’Day S.J., Eggermont A.M., Chiarion-Sileni V., Kefford R., Grob J.J., Mortier L., Robert C., Schachter J., Testori A., Mackiewicz J. (2013). Final results of phase III SYMMETRY study: Randomized, double-blind trial of elesclomol plus paclitaxel versus paclitaxel alone as treatment for chemotherapy-naive patients with advanced melanoma. J. Clin. Oncol..

[212] Platzbecker U., Ades L., Montesinos P., Ammatuna E., Fenaux P., Baldus C., Bernardi M., Berthon C., Bocchia M., Bonmati C. (2025). Arsenic Trioxide and All-Trans Retinoic Acid Combination Therapy for the Treatment of High-Risk Acute Promyelocytic Leukemia: Results From the APOLLO Trial. J. Clin. Oncol..

[213] Zhao H., Fu W., Yang X., Zhang W., Wu S., Ma J., Zhang T., Yao H., Zhang Z. (2026). High-dose vitamin C: A promising anti-tumor agent, insight from mechanisms, clinical research, and challenges. Genes Dis..

[214] Bodeker K.L., Smith B.J., Berg D.J., Chandrasekharan C., Sharif S., Fei N., Vollstedt S., Brown H., Chandler M., Lorack A. (2024). A randomized trial of pharmacological ascorbate, gemcitabine, and nab-paclitaxel for metastatic pancreatic cancer. Redox Biol..

[215] Krishna S., Ganapathi S., Ster I.C., Saeed M.E., Cowan M., Finlayson C., Kovacsevics H., Jansen H., Kremsner P.G., Efferth T. (2015). A Randomised, Double Blind, Placebo-Controlled Pilot Study of Oral Artesunate Therapy for Colorectal Cancer. eBioMedicine.

[216] Invernizzi P., Carbone M., Jones D., Levy C., Little N., Wiesel P., Nevens F. (2023). Setanaxib, a first-in-class selective NADPH oxidase 1/4 inhibitor for primary biliary cholangitis: A randomized, placebo-controlled, phase 2 trial. Liver Int..

[217] Zhu L., Zhao Y., Liu T., Chen M., Qian W.P., Jiang B., Barwick B.G., Zhang L., Styblo T.M., Li X. (2022). Inhibition of NADPH Oxidase-ROS Signal using Hyaluronic Acid Nanoparticles for Overcoming Radioresistance in Cancer Therapy. ACS Nano.

[218] Correia J.H., Rodrigues J.A., Pimenta S., Dong T., Yang Z. (2021). Photodynamic Therapy Review: Principles, Photosensitizers, Applications, and Future Directions. Pharmaceutics.

[219] Wang Y., Wang Q., Wang X., Yao P., Dai Q., Qi X., Yang M., Zhang X., Huang R., Yang J. (2023). Docetaxel-loaded pH/ROS dual-responsive nanoparticles with self-supplied ROS for inhibiting metastasis and enhancing immunotherapy of breast cancer. J. Nanobiotechnology.

[220] Chakrabarti K.R., Hessler L., Bhandary L., Martin S.S. (2015). Molecular Pathways: New Signaling Considerations When Targeting Cytoskeletal Balance to Reduce Tumor Growth. Clin. Cancer Res..

[221] National Library of Medicine National Center for Biotechnology Information. https://clinicaltrials.gov/.

