# Supplementary material for: The Pro-Metastatic Roles of ROS
Source: Antioxidants (Basel). 2026 Apr 22;15(5):529. doi: 10.3390/antiox15050529 (PMC13203640; doi:10.3390/antiox15050529)
Supplement: Supplementary file 1 [file antioxidants-15-00529-s001.zip › antioxidants-4205857-supplementary.pdf]

Supplementary Table S1: Characteristics of Additional ROS Species

ROS species	Main enzymatic / chemical sources	Steady-state levels and lifetime (physiological conditions)	Dominant signaling roles	Dominant damaging roles
**Nitric oxide (*NO)**	Nitric oxide synthases (eNOS, nNOS, iNOS)<br>Nonenzymatic nitrite reduction under hypoxia<br>Some mitochondrial and bacterial sources	Low nanomolar, often 1-100nM<br>Free NO: ~0.1–2s depending on O_2 tension, heme protein and superoxide levels	Cytosol/plasma membrane: classical 2nd messenger regulating vascular tone, neurotransmission, and platelet function<br>Mitochondria: reversible inhibition of respiratory complexes and modulation of bioenergetics and hypoxia signaling<br>Extracellular/vascular: paracrine control of smooth muscle relaxation and leukocyte–endothelium interactions	Cytosol/mitochondria: at high or sustained flux, formation of peroxynitrite with superoxide leads to DNA damage, protein nitration, and mitochondrial dysfunction<br>Proteins: S-nitrosation and metal-center modification become deleterious when widespread, causing enzyme inhibition and dysregulated signaling<br>Tissues: contribution to inflammatory and neurodegenerative injury via nitrosative stress
**Lipid radicals**	Hydrogen abstraction from polyunsaturated fatty acids by hydroxyl radical, peroxynitrite-derived radicals, or high-energy oxidants (including $^1O_2$)<br>Propagation of lipid peroxidation chains in membranes and lipoproteins<br>Iron-catalyzed initiation in ferroptosis	Steady-state bulk concentrations are not well defined; often treated as very low sub-nM to nM<br>Carbon-centered lipid radicals: ns-µs<br>Lipid peroxyl radicals: µs-ms	Membranes: production of downstream electrophilic lipid species that covalently modify Cys/Lys/His in signaling proteins, modulating inflammation, stress responses, and adaptation programs<br>Nucleus/cytosol: lipid-derived signals influence transcription factors via adduct formation	Membranes/organelles: chain lipid peroxidation causing loss of barrier function, altered fluidity, ferroptotic or necrotic cell death<br>Lipoproteins and extracellular milieu: oxidation of LDL and other lipoproteins drive atherogenesis and vascular inflammation<br>Adjacent macromolecules: secondary aldehydes and radical species form adducts with DNA and proteins, contributing to mutagenesis and protein dysfunction
**Singlet oxygen ($^1O_2$)**	Type II photochemical reactions: energy transfer from photoexcited sensitizers to ground-state O_2 in skin, eye, and photodynamically treated tissues, but can be generated during PDT	No measurable steady -state pool<br>Intracellular lifetimes: ~10^{-7}-10^{-6}s	Plasma membrane: localized generation (e.g., during PDT) oxidizes membrane lipids and proteins, leading to altered receptor function and activation of stress and death pathways<br>Cytosol/mitochondria: induces oxidative modifications activate MAPKs, stress kinases, and transcriptional stress responses, acting as a trigger for apoptosis or other stress responses rather than a classical long-range second messenger	Membranes preferential reaction with double bonds in polyunsaturated fatty acids causing rapid lipid peroxidation, loss of membrane integrity, disruption of mitochondrial function, lysosomal permeabilization, and organelle damage central to PDT cytotoxicity<br>Proteins: oxidation of His, Met, Trp and other residues in membrane and nearby proteins results in enzyme/receptor inactivation and structural damage<br>Nucleus/DNA: can

			Extracellular/lipoproteins: contributes to oxidation of LDL and other lipoproteins that influence vascular and inflammatory signaling	oxidize guanine and other bases and contribute to strand breaks promoting mutagenesis and cell death<br>Tissues: generated by photosensitizers and contributes to phototoxicity, tissue injury, and, in therapeutic contexts, tumor ablation and vascular shutdown
**Peroxynitrite ($ONOO^-$)**	Diffusion-controlled reaction of NO with superoxide from NOX, mitochondria, or uncoupled NOS; formed in cytosol, mitochondria, and extracellular milieu	Endogenous steady-state intracellular concentrations are predicted by modeling and are typically in the low nanomolar range.<br>Biological half-life 10^{-3} - 10^{-2}s	Cytosol/mitochondria: modulates signaling by nitration/oxidation of Tyr and Cys residues in kinases, phosphatases, and mitochondrial enzymes, thereby tuning inflammatory and metabolic pathways<br>Extracellular: influences vascular tone and immune responses	DNA: strand breaks and base damage, triggering PARP activation and energy failure<br>Proteins: 3-nitrotyrosine formation, Cys/Met oxidation, inactivation of mitochondrial and cytosolic enzymes<br>Lipids: lipid peroxidation and membrane disruption
**Hypochlorous acid (HOCl)**	Myeloperoxidase-catalyzed reaction of H_2O_2 with Cl^- in neutrophils and some monocytes	Usually absent or extremely low except in activated myeloid cells.<br>Very short < ms	Extracellular / phagosomal: contributes to microbicidal activity of neutrophils; modifies microbial and host surface proteins, altering receptor signaling and cytokine milieu<br>Tissues: low, chronic HOCl can modulate redox-sensitive pathways	Extracellular matrix and plasma: chlorination/ oxidation of proteins, disruption of matrix structure and lipoprotein modification<br>Cells at inflammatory sites: oxidation and chlorination of Cys, Met, and amine groups, enzyme inactivation, membrane lipid oxidation, and promotion of necrosis/ apoptosis
